# HP1a-mediated heterochromatin formation promotes antimicrobial responses against *Pseudomonas aeruginosa* infection

**DOI:** 10.1186/s12915-022-01435-8

**Published:** 2022-10-20

**Authors:** Po-Jen Wu, Shian-Jang Yan

**Affiliations:** 1 Institute of Basic Medical Sciences, College of Medicine, National Cheng-Kung University, No. 1, University Road, Tainan City, Taiwan; 2 Department of Physiology, College of Medicine, National Cheng-Kung University, No. 1, University Road, Tainan City, Taiwan

**Keywords:** Heterochromatin protein 1a (HP1a), Heterochromatin formation, *Pseudomonas aeruginosa*, Fat body, Antimicrobial responses

## Abstract

**Background:**

*Pseudomonas aeruginosa* is a Gram-negative bacterium that causes severe infectious disease in diverse host organisms, including humans. Effective therapeutic options for *P. aeruginosa* infection are limited due to increasing multidrug resistance and it is therefore critical to understand the regulation of host innate immune responses to guide development of effective therapeutic options. The epigenetic mechanisms by which hosts regulate their antimicrobial responses against *P. aeruginosa* infection remain unclear. Here, we used *Drosophila melanogaster* to investigate the role of heterochromatin protein 1a (HP1a), a key epigenetic regulator, and its mediation of heterochromatin formation in antimicrobial responses against PA14, a highly virulent *P. aeruginosa* strain.

**Results:**

Animals with decreased heterochromatin levels showed less resistance to *P. aeruginosa* infection. In contrast, flies with increased heterochromatin formation, either in the whole organism or specifically in the fat body—an organ important in humoral immune response—showed greater resistance to *P. aeruginosa* infection, as demonstrated by increased host survival and reduced bacterial load. Increased heterochromatin formation in the fat body promoted the antimicrobial responses via upregulation of fat body immune deficiency (imd) pathway-mediated antimicrobial peptides (AMPs) before and in the middle stage of *P. aeruginosa* infection. The fat body AMPs were required to elicit HP1a-mediated antimicrobial responses against *P. aeruginosa* infection. Moreover, the levels of heterochromatin in the fat body were downregulated in the early stage, but upregulated in the middle stage, of *P. aeruginosa* infection.

**Conclusions:**

These data indicate that HP1a-mediated heterochromatin formation in the fat body promotes antimicrobial responses by epigenetically upregulating AMPs of the imd pathway. Our study provides novel molecular, cellular, and organismal insights into new epigenetic strategies targeting heterochromatin that have the potential to combat *P. aeruginosa* infection.

**Supplementary Information:**

The online version contains supplementary material available at 10.1186/s12915-022-01435-8.

## Background

The widely distributed Gram-negative bacteria *Pseudomonas aeruginosa* causes infections in diverse host organisms [1]. It is an opportunistic pathogen and major cause of human infection and death in hospitals due to its high virulence and multidrug resistance (MDR) [2, 3]. The serious infections caused by *P. aeruginosa* occur mainly in severely ill and immunocompromised patients [4, 5]. However, *P. aeruginosa* rarely causes severe infection in normal, immunocompetent hosts, suggesting that antimicrobial responses play a critical role in resistance to *P. aeruginosa* infection [6]. Antimicrobial responses involve innate immune activation, which is responsible for early recognition of invading pathogens and their clearance [7]. The innate immune cells are first responders that are activated by pathogens, such as bacteria and viruses. In response to bacterial infection, the activated innate immune cells produce antimicrobial peptides (AMPs) that eliminate the pathogens. Notably, the natural AMPs act as barriers that protect human skin and airway epithelium from bacterial infection [8, 9] and have drawn great interest as targets in the development of drugs to fight MDR bacteria, including *P. aeruginosa* [10, 11]. It is therefore critical to understand the regulation of host innate immune responses to *P. aeruginosa* infection as a guide in development of effective therapeutic options [12].

Innate immune responses are evolutionarily conserved from invertebrates to humans and are dependent upon conserved signaling cascades and expression of innate immune responsive genes [13]. Gene expression is epigenetically controlled by chromatin state, which is also a conserved mechanism in higher eukaryotes, while growing evidence supports a role of chromatin state in the regulation of innate immune responsive gene transcription [14, 15]. Posttranslational modification of histone tails plays a major role in epigenetic gene regulation by mediating the transition of chromatin between the loose euchromatin and compact heterochromatin states [16–18]. Histone 3 lysine 9 di/tri-methylation (H3K9me2/3) marks a major type of heterochromatin with the binding of their reader, heterochromatin protein 1a (HP1a), to promote heterochromatin formation [19–23]. HP1a can form bridges between nucleosomes [24–26] and recruit the writer, H3K9 methyltransferases, to methylate H3K9 [27]; these processes further promote heterochromatic propagation [17]. Thus, HP1a protein and H3K9me2/me3 modification contribute to heterochromatin formation and maintenance. Recent evidence showed that heterochromatin formation participates in cellular immune responses, such as macrophage activation [28] and cancer cells with innate immune activation [29]. Several studies have demonstrated that heterochromatin contributes to immune responses of innate immune cells via H3K9 methylation of immune responsive genes [30–32]. However, the molecular and cellular mechanisms by which HP1a-mediated heterochromatin formation regulates innate immune responses against *P. aeruginosa* infection remain elusive.

The model organism *Drosophila melanogaster* plays a critical role in studies of human innate immunity. *Drosophila* have similar innate immune pathways to human NF-kB signaling, including immune deficiency (imd) and Toll, which initiate the cascade of innate immune responses [33]. The imd pathway, which includes many kinds of peptidoglycan recognition proteins (PGRP), is important in antimicrobial responses to Gram-negative bacteria, while the Toll pathway responds preferentially to Gram-positive bacteria [34]. The cascades of the imd and Toll pathways mediate nuclear import of NF-kB homologs, which upregulate expression of AMPs that protect against invading bacteria [33, 35]. Suppression of those AMPs increases the susceptibility of *Drosophila* to virulent *P. aeruginosa* infection [36, 37]. The *Drosophila* fat body is the major immune organ active in humoral responses involving secretion of AMPs, and it is activated by imd and Toll signaling upon systemic bacterial infection [38]. Thus, it raises the interesting question of whether HP1a-mediated heterochromatin formation in the fat body regulates innate immune responses against *P. aeruginosa* infection.

To determine the role of HP1a-mediated heterochromatin formation in innate immune responses, we manipulated heterochromatin levels through whole body overexpression of *HP1a* to elicit gain of function, by using *HP1a* mutant flies that demonstrate loss of *HP1a* function, or through overexpression of *HP1a* specifically in the fat body. These experimental models were used to investigate the role of heterochromatin levels in antimicrobial responses upon systemic *P. aeruginosa* PA14 infection. Our results demonstrate that elevated HP1a-mediated heterochromatin formation in the fat body increases survival and decreases bacterial loads upon *P. aeruginosa* infection. Mechanistically, we found that fat body heterochromatin formation promotes the upregulation of imd pathway-mediated AMPs before, and in the middle stage of, *P. aeruginosa* infection. Importantly, the imd pathway-mediated AMPs, including Diptericin A (DptA), were required to support HP1a-mediated antimicrobial responses after *P. aeruginosa* infection. Furthermore, during *P. aeruginosa* infection, an early stage decrease in fat body heterochromatin levels was observed, followed by increased levels in the middle stage. These results provide molecular, cellular, and organismal level insights regarding HP1a and heterochromatin mediated epigenetic control of antimicrobial responses against *P. aeruginosa* infection.

## Results

### Increased formation of heterochromatin in the whole organism or the fat body of *Drosophila* promotes resistance to *P. aeruginosa* infection

**Fig. 1:**
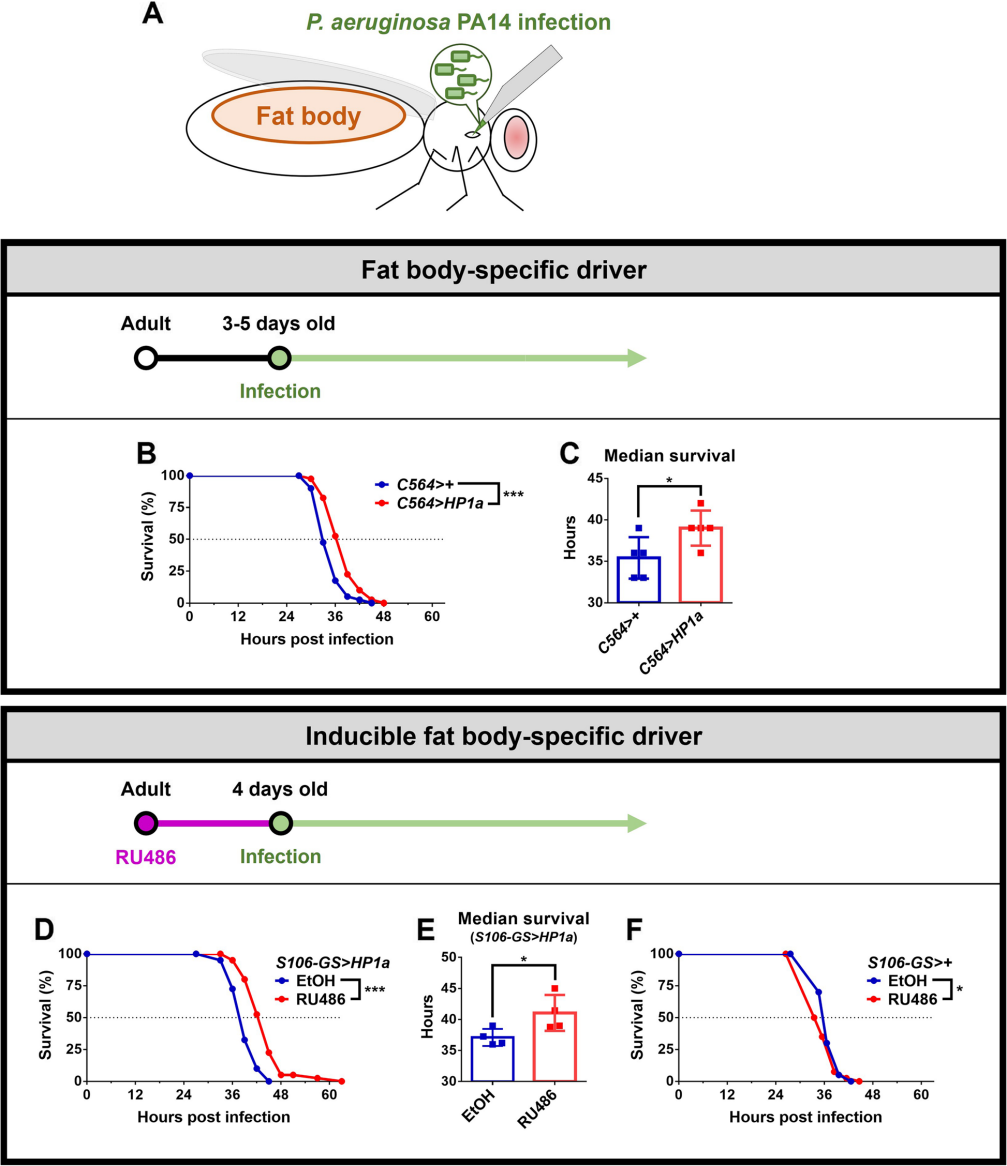
Increased HP1a-mediated heterochromatin formation in the fat body promotes resistance against systemic *P*. *aeruginosa* PA14 infection. **A** Systemic *P*. *aeruginosa* PA14 infection was achieved through surgical wound via needle pricking of the *Drosophila* thorax. The immune organ (fat body) is located in the abdomen. **B**, **C** The survival curve and median survival of flies with fat body-specific *C564*-driven *HP1a* overexpression, compared to control, after PA14 infection. **D**, **E** The survival curve and median survival of flies with RU486-inducible fat body specific HP1a overexpression after PA14 infection. Control and treated flies received food with either EtOH (vehicle) or RU486, respectively, for 4 days upon eclosion. **F** Exposure to RU486 did not reduce, but rather increased, susceptibility and reduced survival of *S106-GS>+* expressing flies after PA14 infection. For survival curves, *n* = 40 flies/group and *p < 0.05, ***p < 0.001 by log-rank test. For median survival, data are shown as mean ± SD from 4-5 independent experiments and *p < 0.05 by Student’s *t*-test. Inoculum of OD600 = 0.1

To establish a *Drosophila*-based platform for exploring antimicrobial responses to *P. aeruginosa* infection, we used the surgical wound method to conduct infection of adult male wild type (WT) flies with *P. aeruginosa* PA14 at 3-5 days post-eclosion, which caused inoculum-dependent lethality (Additional file 1: Fig. S1A). Importantly, flies with ubiquitous induction of heterochromatin formation via overexpression of *HP1a* showed greater resistance to *P. aeruginosa* versus WT flies, whereas *HP1a* loss-of-function mutant flies, which have decreased heterochromatin levels, were more vulnerable compared to WT (Additional file 1: Fig. S1B, C) [39, 40]. The *Drosophila* fat body (Fig. 1A) is the primary organ responsible for immune defense. Therefore, we determined whether local, tissue-specific induction of heterochromatin formation in the fat body would counteract systemic *P. aeruginosa* infection. Survival of flies with fat body-specific overexpression of *HP1a* by *C564* [41], an independent fat body driver, was increased (Fig. 1B, C, Additional file 1: Fig. S2A-D). Moreover, we inoculated flies with lower *P. aeruginosa* doses and found that increased fat body HP1a-mediated heterochromatin promotes host resistance to PA14 infection with a lower bacterial dose (Additional file 1: Fig. S3). However, knockdown of *HP1a* in the fat body did not affect host susceptibility to infection (Additional file 1: Fig. S4A, B). These results suggest that HP1a-mediated heterochromatin formation improves host resistance against *P. aeruginosa*, and increased heterochromatin formation in fat body tissue alone is sufficient to promote host resistance.

To eliminate the confounding factors due to genetic background variability in fly strains, we used the inducible Gene Switch (GS) system for conditional fat body-specific *HP1a* overexpression. As shown in Additional file 1: Fig. S5, the green fluorescent protein (GFP) signal was prominently detected in the abdomen of flies carrying the fat body-specific *S106-GS Gal4* driver [42] in combination with the UAS-nuclear GFP transgene after receiving food containing 600 μM RU486. These data demonstrate effective and location-specific induction of the fat body driver-linked gene expression. Temporal overexpression of *HP1a* in the fat body via this RU486-inducible expression system conferred elevated host resistance against *P. aeruginosa* PA14 infection (Fig. 1D, E, Additional file 1: Fig. S2E-G). Moreover, dietary exposure to RU486 did not promote resistance, but rather increased vulnerability of the control flies (i.e., the progeny of the *S106-GS Gal4* driver line crossed to the *w*^*1118*^ strain) to *P. aeruginosa* infection (Fig. 1F). Since *Drosophila* do not possess acquired/adaptive immune responses, these results suggest that upregulating the level of heterochromatin formation in the host genome could increase the effectiveness of innate defense responses. Taken together, our data demonstrate that heterochromatin formation, especially in the fat body, plays an important role in promoting resistance to *P. aeruginosa* infection.

### Increased heterochromatin formation in the fat body reduces *P. aeruginosa* loads within infected adult flies

**Fig. 2:**
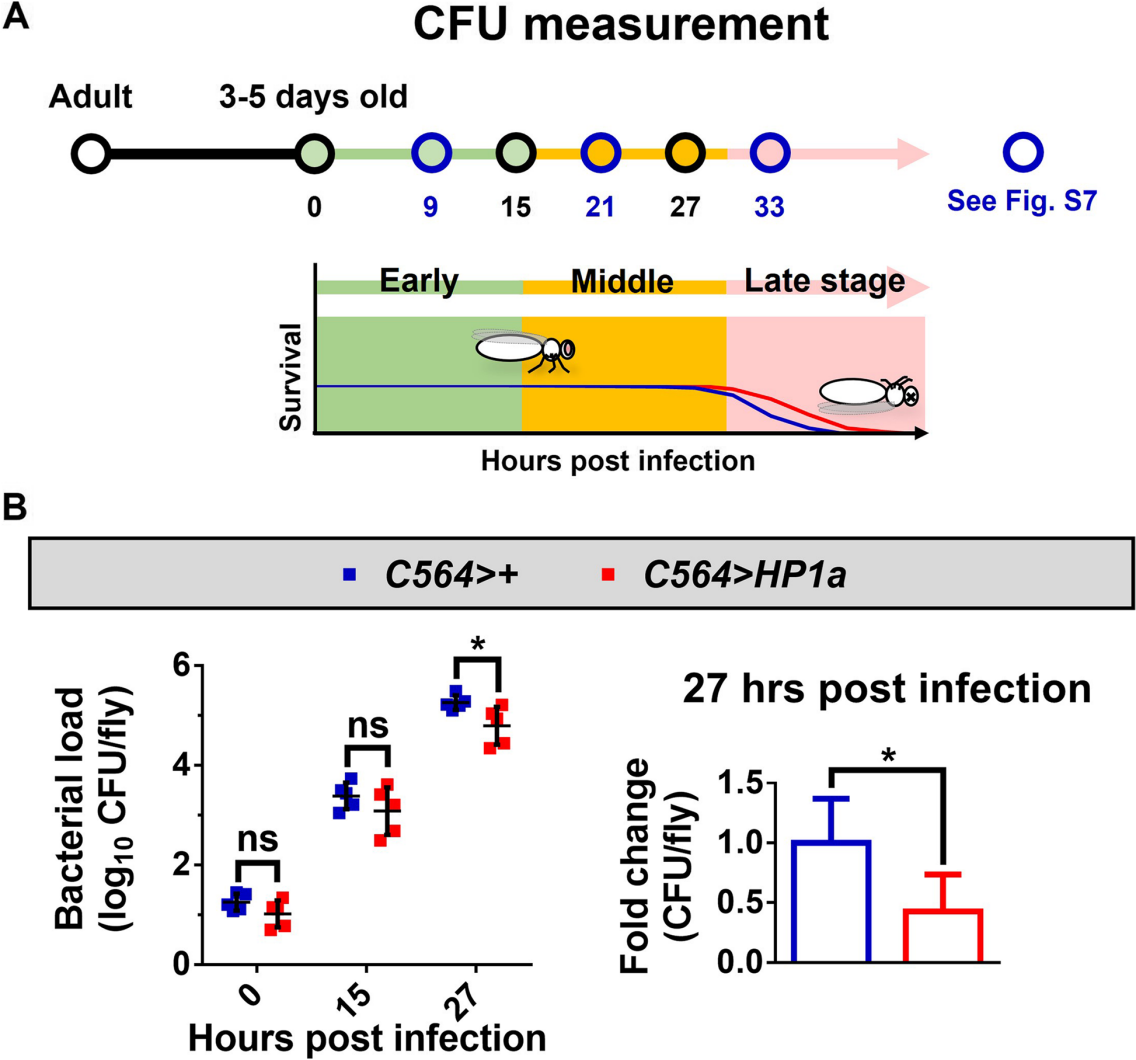
Increased fat body heterochromatin formation reduces the bacterial loads of *P*. *aeruginosa*. **A** Schematic of fly collection at different time points in the early, middle, and late stages of PA14 infection for the CFU measurement. CFU results for time points indicated by blue circles are presented in Fig. S5. **B** Bacterial loads of *C564*-driven *HP1a* and control flies were counted at 0, 15, and 27 hpi. CFU experiments at 15 and 27 hpi are representative of 5 independent experiments, and each experiment included 5 flies for the CFU measurement. CFU experiments at 0 hpi are representative of 5 independent experiments, and each experiment included 1 fly for the CFU measurement. *p < 0.05 by Student’s *t*-test. CFU, colony forming unit; hpi, hrs post infection; ns, not significant

The course of *P. aeruginosa* infection is divided into early (0–15 h post infection [hpi]), middle (15–30 hpi), and late (beyond 30 hpi) stages based on the survival curve, as depicted in Fig. 2A. The flies began dying at 30 hpi (used to determine the middle and late stages) with the halfway time point set at 15 hpi (used to determine the early and middle stages). Since the *Drosophila* fat body is the main immune response tissue that secretes AMPs for the elimination of invading bacteria, we sought to determine the bacterial clearance capacity arising from fat body-specific *HP1a* overexpression by monitoring the viable bacterial load within the flies, recorded as colony-forming units (CFU) per fly at different time points: 0, 9, 15, 21, 27, and 33 h after challenge with *P. aeruginosa* PA14. Bacterial loads/CFU counts of *C564*-driven *HP1a* flies showed significant clearance and were decreased by 57% at 27 hpi (Fig. 2B). Moreover, consistent with our findings in Fig. 1B, we found that fat body-specific overexpression of *HP1a* using an alternative fat body driver, *Lsp2* [43], also increased survival after *P. aeruginosa* infection (Additional file 1: Fig. S6A-F) and decreased bacterial loads by 86% at 27 hpi compared to control flies (Additional file 1: Fig. S6G). It is noteworthy that increased HP1a-mediated heterochromatin formation promoted bacterial clearance at 27 hpi (Fig. 2B), before the middle-to-late stage transition of *P. aeruginosa* infection, but not earlier (at 21 hpi) in the middle stage or after (at 33 hpi) in the late stage of the infection (Additional file 1: Fig. S7). Together, these results suggest that increased fat body heterochromatin formation reduces the bacterial load associated with *P. aeruginosa* infection.

### Increased heterochromatin formation in cells of the *Drosophila* fat body promotes imd-mediated antimicrobial responses against *P. aeruginosa* infection

**Fig. 3:**
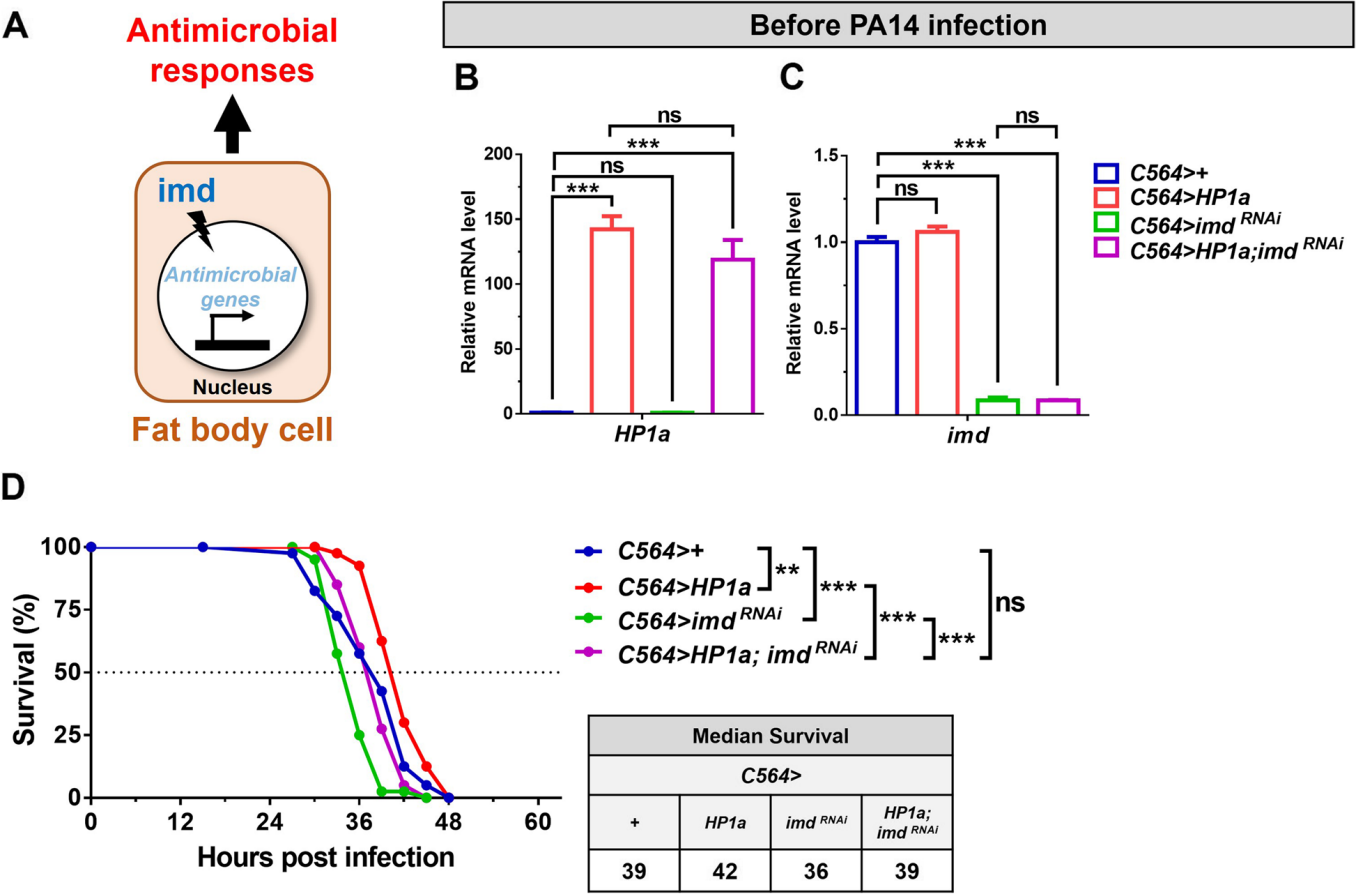
The imd-mediated antimicrobial response in the *Drosophila* fat body is necessary for heterochromatin-mediated resistance to *P*. *aeruginosa* infection. **A** The fat body promotes antimicrobial responses through the imd pathway upon infection by Gram-negative bacteria. **B**, **C** Relative mRNA expression in the fat body before PA14 infection. *n* = 8 flies/group. Data are shown as mean ± SD and ***p < 0.001 by Student’s *t*-test. **D** The survival curve and median survival in flies with fat body-specific *C564*-driven +, *HP1a*, *imd* ^*RNAi*^, and *HP1a; imd* ^*RNAi*^ after PA14 infection. *n* = 40 flies/group. **p < 0.01, ***p < 0.001 by log-rank test. ns, not significant

To further determine the molecular mechanisms by which HP1a-mediated heterochromatin formation regulates innate immune responses, we examined whether the imd pathway, which is critical for antimicrobial responses to Gram-negative bacteria, participates in the fat body heterochromatin formation-induced antimicrobial immune response against Gram-negative *P. aeruginosa* PA14 infection (Fig. 3A). We first confirmed that fat body cell *HP1a* mRNA is upregulated by 150-fold in flies with *C564*-driven *HP1a* and *HP1a; imd*^*RNAi*^ compared to control flies and that fat body cell *imd* mRNA is downregulated by 90% in flies with *C564*-driven *imd*^*RNAi*^ and *HP1a; imd*^*RNAi*^ compared to control flies (Fig. 3B, C). Importantly, *HP1a* and *imd* mRNA levels were not affected in the *C564>imd*^*RNAi*^ and *C564>HP1a* flies, respectively, compared to control flies. These results suggest that overexpression and knockdown of *HP1a* and *imd*, respectively, work effectively and specifically in the fat body. Next, we investigated the host resistance in the flies with fat body-specific *C564*-driven *+*, *HP1a*, *imd*^*RNAi*^, and *HP1a; imd*^*RNAi*^ after *P. aeruginosa* infection. Flies with increased fat body heterochromatin formation by *HP1a* overexpression had consistently increased resistance to *P. aeruginosa* infection compared to controls, and hypoactivation of the imd pathway by *imd* downregulation decreased that resistance (Fig. 3D). While *imd* downregulation in the fat body impaired heterochromatin formation-induced antimicrobial responses, increased fat body cell heterochromatin formation in the flies with *imd* knockdown restored the host resistance to *P. aeruginosa* infection to the same level observed in *C564*-driven + controls. Together, these results suggest that HP1a-mediated heterochromatin formation promotes imd-mediated antimicrobial responses against *P. aeruginosa* infection.

**Fig. 4:**
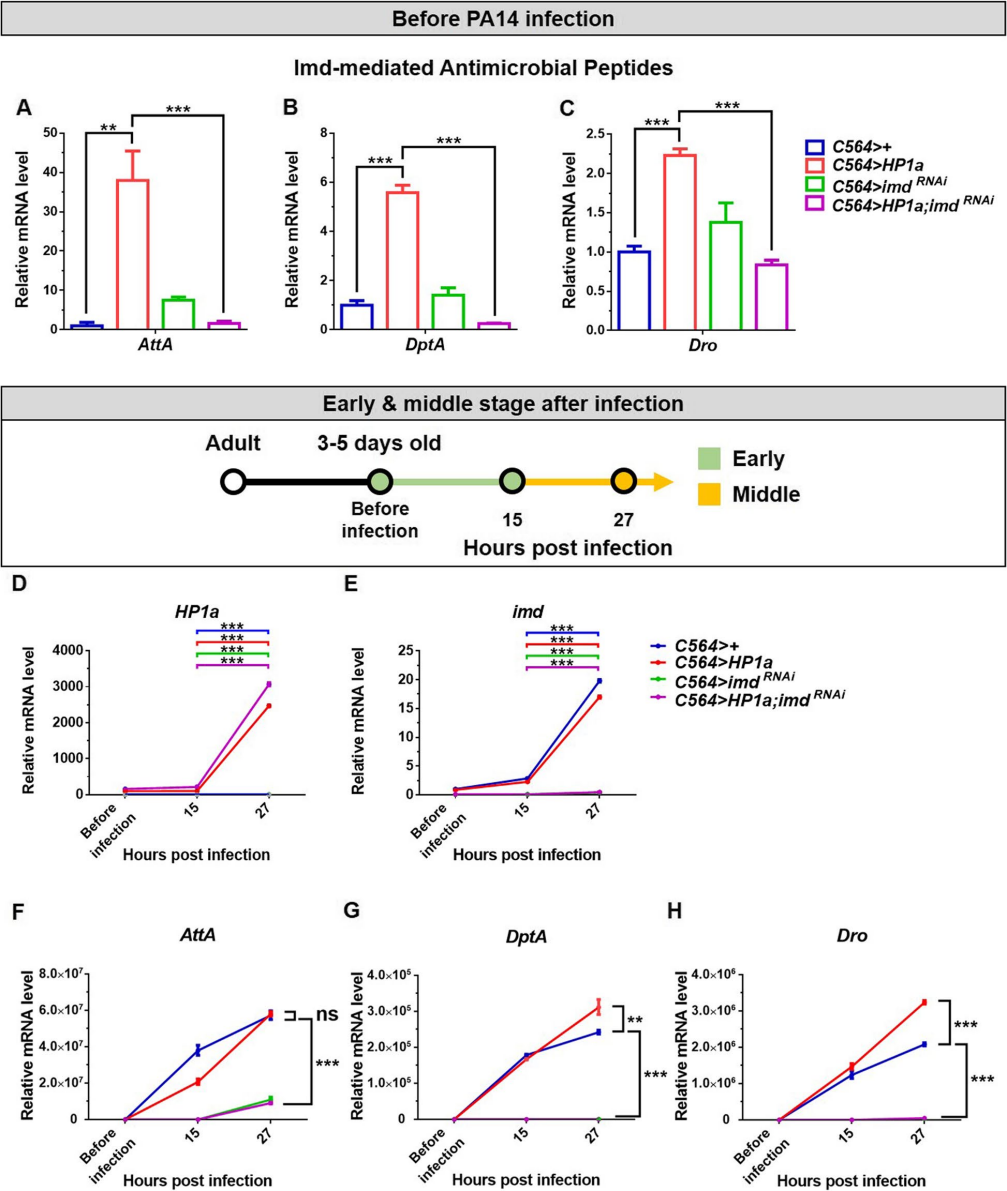
Increased heterochromatin formation in the fat body promotes imd pathway mediated DptA and Dro overexpression. **A**–**C** Relative expression of imd-mediated AMP mRNA in the fat body was measured before PA14 infection. *n*= 8 flies/group. **D**–**H** Relative expression of *HP1a*, *imd*, *DptA*, *Dro*, and *AttA* mRNA in the fat body was measured in the early and middle stages of PA14 infection. Note that *C564>HP1a* flies already showed constitutive upregulation of various AMP as compared to control *C564>+* before infection as shown in **A**–**C** but the differences are not easily observed in **D**–**H** due to the log scale. n=4 flies/group at each time point. Data are shown as mean ± SD and **p < 0.01, ***p < 0.001 by Student’s *t*-test. ns, not significant

The imd pathway is required for AMP gene expression in response to Gram-negative bacteria. Therefore, we next asked whether increased fat body heterochromatin formation activates the expression of imd-mediated AMPs. We found that, even before infection, increased heterochromatin formation was associated with specific upregulation of several imd-mediated AMPs (Additional file 1: Fig. S8A). Moreover, the *HP1a* overexpression-induced *imd*-mediated AMPs, including *Attacin A* (*AttA*), *Diptericin* (*DptA*), and *Drosocin* (*Dro*), were not only upregulated to the greatest extent when heterochromatin was increased in the fat body (Fig. 4A–C, Additional file 1: Fig. S8A) but were also downregulated most significantly by simultaneous knockdown of *imd* (Fig. 4A–C). Interestingly, we found that *HP1a* and *imd* mRNA in the fat body are drastically upregulated in all flies at 27 hpi compared to 15 hpi, while fat body-specific *C564*-driven + and *HP1a* groups express higher *imd* levels than the corresponding groups expressing *imd*^*RNAi*^ and *HP1a; imd*^*RNAi*^ (Fig. 4D, E, Additional file 1: Fig. S9A, B, F, G). These data suggest that HP1a-mediated heterochromatin formation regulates antimicrobial immune responses via the imd pathway in the *Drosophila* fat body upon *P. aeruginosa* infection. Next, we determined whether HP1a-mediated heterochromatin regulates other immune or stress responses such as the Toll and JAK-STAT pathways to activate antibacterial activity. Before infection, the expression of Toll-mediated AMPs in the fat body with increased heterochromatin levels showed that *Bomanin 1 (BomS1)*, *Drosomycin (Drs)*, *and Daisho1 (Dso1)* are upregulated and that *Baramicin (Bara)* and *Daisho2 (Dso2)* are downregulated (Additional file 1: Fig. S8A). These results suggest that HP1a-mediated heterochromatin selectively regulates expression of Toll-mediated AMPs. Moreover, the JAK-STAT response genes including *Turandot A (TotA)* and *STAT92E* were not upregulated by increased fat body HP1a-mediated heterochromatin formation, suggesting that HP1a overexpression does not induce a major stress before infection (Additional file 1: Fig. S8B). Additionally, after infection, *AttA*, *DptA*, and *Dro* were all drastically upregulated at 27 hpi, while the *HP1a* overexpressing animals expressed even higher levels of *DptA* and *Dro* than control (Fig. 4F–H, Additional file 1: Fig. S9C-E, H-J). These results indicate that increased heterochromatin formation in the fat body promotes upregulation of imd-mediated AMPs in the middle stage of *P. aeruginosa* infection.

### Fat body DptA is sufficient and necessary for HP1a-mediated antimicrobial responses against *P. aeruginosa* infection

**Fig. 5:**
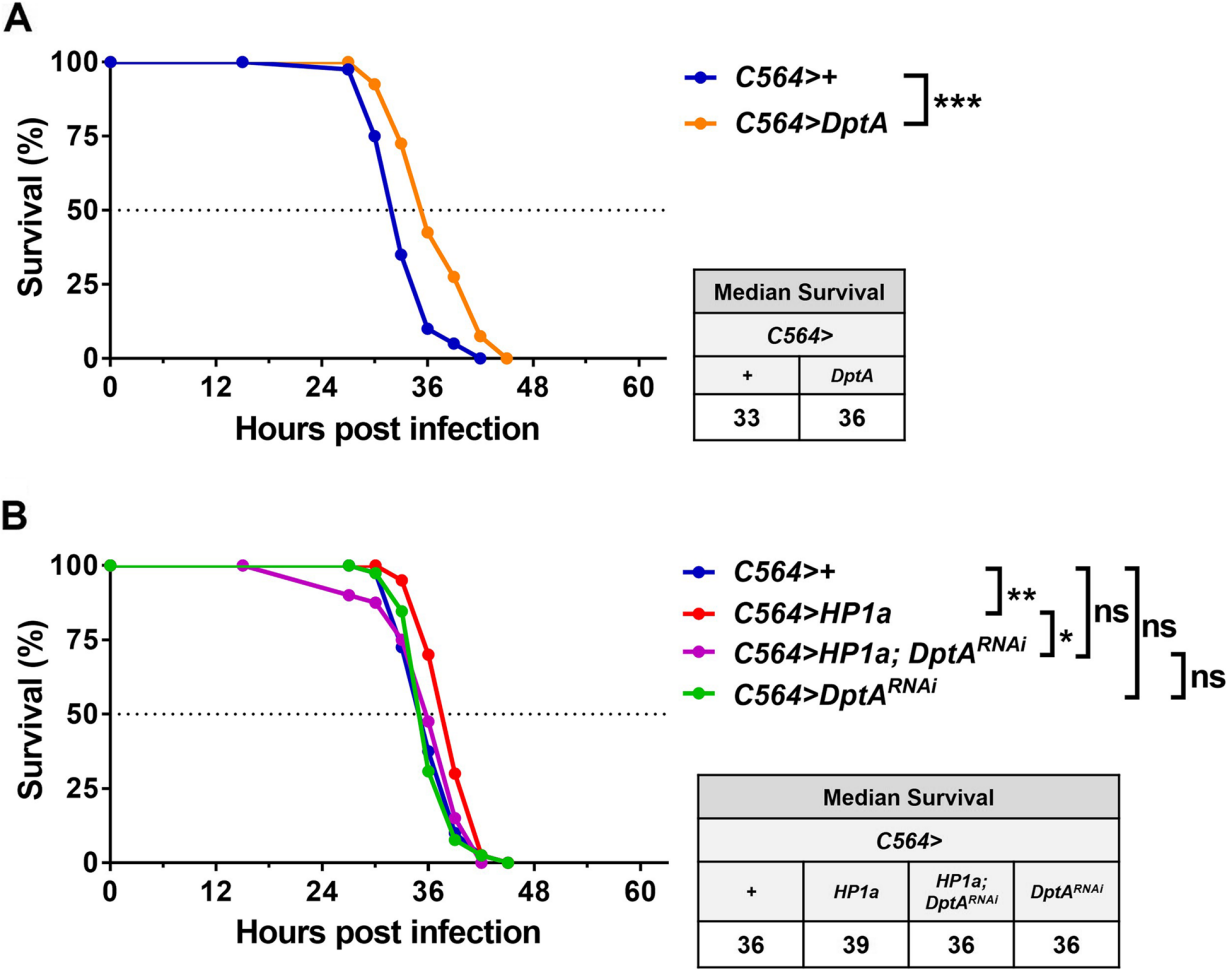
Increased DptA expression in the fat body is sufficient and necessary for HP1a-mediated antimicrobial responses against PA14 infection. **A** The survival curve and median survival in flies with fat body-specific *C564*-driven + and *DptA* expression after PA14 infection. **B** The survival curve and median survival in flies with fat body-specific *C564*-driven +, *HP1a*, *DptA*^*RNAi*^, and *HP1a; DptA*^*RNAi*^ expression after PA14 infection. *n* = 40/group. *p < 0.05, **p < 0.01, ***p < 0.001 by logrank test. ns, not significant

To determine whether the AMPs, including *AttA*, *DptA*, and *Dro*, are required for host resistance to *P. aeruginosa* infection, we examined survival in flies with fat body-specific overexpression or knockdown of these AMPs after infection. Fat body *DptA* and *Dro* mRNA were expressed at > 900 and > 15,000 fold, respectively, in *C564*>*DptA* and *C564>Dro* flies compared to controls (Additional file 1: Fig. S10A, B). *DptA* overexpression in the *Drosophila* fat body was associated with increased resistance to *P. aeruginosa* infection and longer survival compared to controls (Fig. 5A). Moreover, knockdown of *DptA* in the fat body decreased resistance to *P. aeruginosa* infection after induction by increased fat body HP1a-mediated heterochromatin formation (Fig. 5B). In contrast, *Dro* overexpression in the fat body alone did not lead to increased resistance to *P. aeruginosa* infection (Additional file 1: Fig. S10C), suggesting that HP1a-mediated heterochromatin formation-induced *Dro* upregulation is not sufficient to promote resistance to *P. aeruginosa* infection. Likewise, *AttA* was not necessary to promote resistance to *P. aeruginosa* infection in flies with increased fat body heterochromatin formation (Additional file 1: Fig. S10D). These results suggest that fat body heterochromatin formation-induced *DptA* upregulation is a key event that is sufficient and necessary to trigger HP1a-mediated antimicrobial responses to *P. aeruginosa* infection.

### Heterochromatin formation in cells of the fat body is required in the middle stage of *P. aeruginosa* infection

**Fig. 6:**
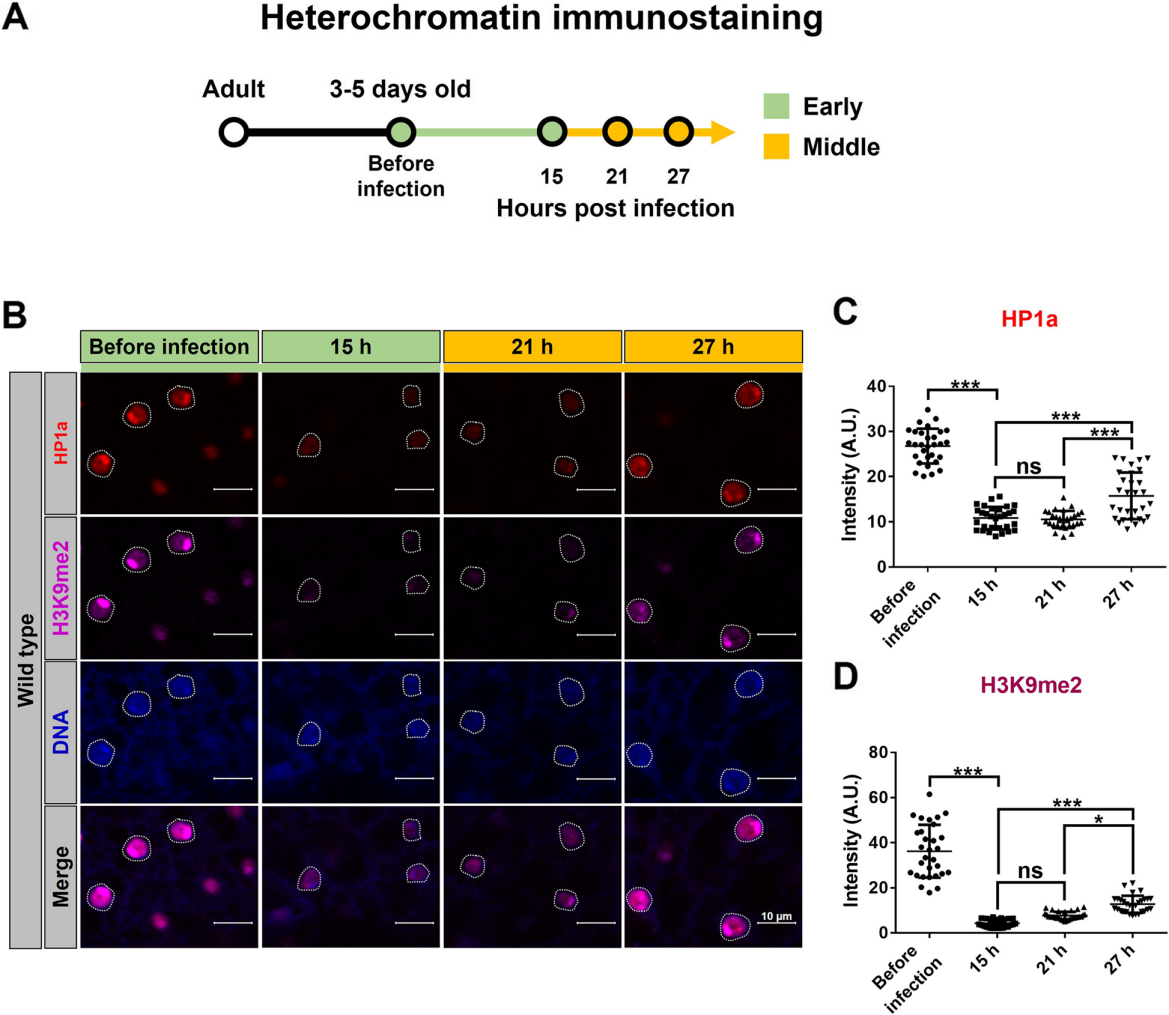
Fat body heterochromatin levels are downregulated in the early stage but upregulated in the middle stage of *P*. *aeruginosa* infection. **A** Schematic of fly collection at different time points of the early and middle stages of PA14 infection for heterochromatin marker immunostaining. **B** Representative images of fat body cells from wild type flies with HP1a (red) and H3K9me2 (magenta) immunostaining, and nuclear DNA (blue) staining with Hochest. Scale bar: 10 μm. **C**, **D** Immunofluorescence intensity of HP1a (**C**) and H3K9me2 (**D**) before infection and at 15, 21, and 27 hpi. All results involved staining of tissue from two flies (*n* = 15/fly). Data are shown as mean ± SD and *p < 0.05, ***p < 0.001 by Student’s *t*-test. hpi, hrs post infection; ns, not significant

We noticed that *HP1a* mRNA was upregulated in the middle stage of *P. aeruginosa* infection (Fig. 4D). To further characterize antimicrobial responses associated with HP1a-mediated heterochromatin formation in cells of the *Drosophila* fat body, we performed immunostaining to quantify heterochromatin levels in the early and middle stages of *P. aeruginosa* PA14 infection (Fig. 6A). Heterochromatin levels, indicated via H3K9me2 and HP1a staining, were decreased at 15 hpi compared to levels before infection (Fig. 6B–D). Heterochromatin levels then increased at 27 hpi compared to 15 hpi. These data indicate upregulation of HP1a protein expression, in addition to *HP1a* mRNA, at 27 hpi compared to 15 hpi. Our data demonstrate that fat body heterochromatin levels are decreased in the early stage and increased in the middle stage of *P. aeruginosa* infection. These results suggest that time-dependent upregulation of *Drosophila* fat body heterochromatin formation is required in the middle stage of *P. aeruginosa* infection.

## Discussion

**Fig. 7:**
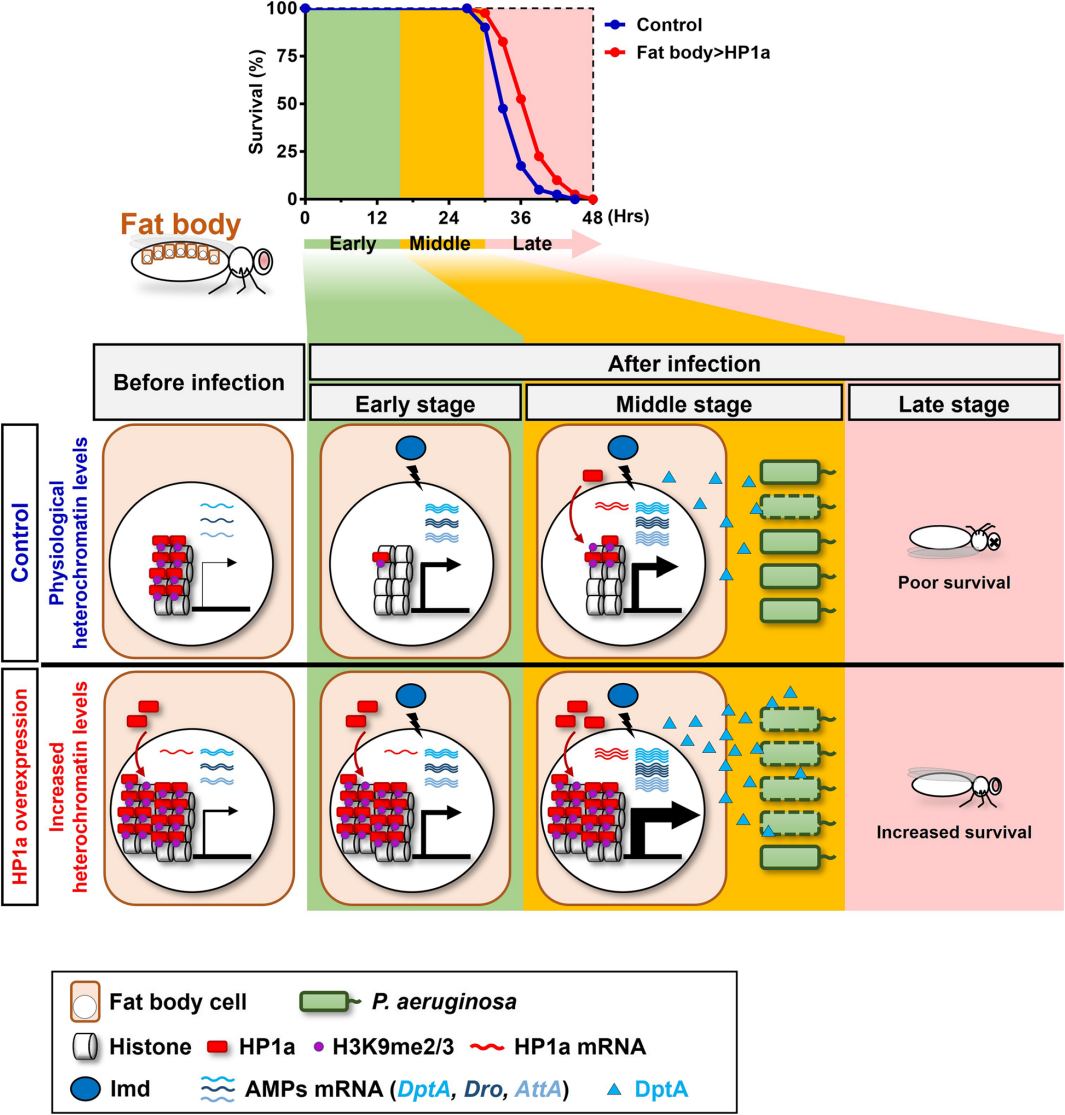
Heterochromatin formation promotes antimicrobial responses through imd pathway mediated expression of AMPs, including DptA. Increased HP1a-mediated heterochromatin formation in the fat body promotes resistance to systemic *P*. *aeruginosa* infection. Before infection, fat body HP1a-mediated heterochromatin formation induces overexpression of imd pathwayrelated AMPs. Mechanistically, upon *P*. *aeruginosa* infection, increased heterochromatin formation in the fat body promotes antimicrobial responses through imd pathway mediated DptA expression. Moreover, upon *P*. *aeruginosa* infection, the time-dependent, physiological heterochromatin levels are tightly regulated via epigenetic changes: downregulated in the early stage of *P*. *aeruginosa* infection and then upregulated in the middle stage. Increased fat body heterochromatin formation improves antimicrobial responses against *P*. *aeruginosa* infection and promotes host survival

Characterization of pathways involved in antimicrobial responses to the highly virulent and MDR bacteria *P. aeruginosa* is a critical pursuit in infectious disease microbiology. Currently, the epigenetic mechanisms of host antimicrobial responses against *P. aeruginosa* infection are poorly understood. To address this important area of research, we employed *Drosophila*, the genetically tractable model organism in which innate immunity was first discovered [44], to determine interactions between *P. aeruginosa* and the host after surgical wound infection. We found that increased HP1a-mediated heterochromatin formation in the fat body, an immune organ that mediates humoral responses, promoted antimicrobial responses, including host resistance to bacterial infection and reduction of bacteria load (Fig. 7). Furthermore, the imd pathway, a key signaling sequence in response to Gram-negative bacteria, and its downstream AMPs, including DptA, were found to mediate fat body heterochromatin formation-induced antimicrobial responses against *P. aeruginosa* infection. We also demonstrated that fat body heterochromatin levels are tightly regulated in timing in response to *P. aeruginosa* infection. Heterochromatin levels decrease in the early stage of infection but then increase in the middle stage. Through our study, we identified a novel epigenetic mechanism in which increased heterochromatin formation in the *Drosophila* fat body promotes antimicrobial responses against *P. aeruginosa* infection.

AMPs are produced as a first-line defense in most cell types [45–47]. AMPs are positively charged, amphipathic peptides that disrupt bacteria through non-specific interactions with the negatively charged bacterial cell membrane [38]. Because of their broad activity, non-specificity, and rapid action, AMPs can limit emergence of bacterial resistance and have been used in many clinical trials [48]. Indeed, overexpression of *DptA* and *AttA* provide protection against *P. aeruginosa* infection in immunocompromised *Drosophila* [37]. In our study, we found that HP1a-mediated antimicrobial responses to *P. aeruginosa* infection in *Drosophila* were dependent on *DptA* expression, but not on *AttA* (Fig. 5, Additional file 1: Fig. S10D), suggesting that DptA is a potent antimicrobial agent. It raises our curiosity whether DptA works alone or together with other AMPs to confer specificity, if any, to host resistance against *P. aeruginosa* infection. To characterize the contribution of DptA and also minimize possible background effects, we performed the *P. aeruginosa* infection in the *Dpt*^*SK1*^ (*DptA* and *DptB* mutations), *∆AMP*^*+Dpt*^ (*AttA-D*, *Def*, *Dro*, *Drs*, and *Mtk* mutations), *∆AMP* (combined 10 AMP mutations), and control iso *w*^*1118*^ flies [49]. Interestingly, *∆AMP* flies were more vulnerable to PA14 infection (Additional file 1: Fig. S11) [49]. However, *Dpt*^*SK1*^ and *∆AMP*^*+Dpt*^ flies had similar host susceptibility to PA14 infection compared to control, iso *w*^*1118*^. These results suggest that *DptA* is not specific for host resistance to *P. aeruginosa* infection and yet has combinatory contribution with *DptB* and other 8 AMPs to resist PA14 infection. Moreover, although upregulation of *Dro* in the fat body is associated with increased HP1a-mediated heterochromatin in the middle stage of *P. aeruginosa* infection, overexpression of *Dro* does not increase host survival upon infection (Additional file 1: Fig. S10C). Previous findings have revealed an incredible specificity for *DptA* in defense against *P. rettgeri* infection [49, 50]. Similar AMP microbe specificities have been shown for *Daisho* and *Fusarium* fungi [51] and a recent study by Hanson et al. shows a Drosocin-derived peptide is specific to *P. burhodogranariea* [52]. Interestingly, an elegant new study by Shaka et al. also found Drosocin mediates susceptibility to *P. alcalifaciens*, specifically according to LPS biosynthesis pathways [53]. Consistent with these previous findings, our results indicate that the additive/synergistic combination and specificity of antimicrobial activity shown by different AMPs are important research areas that require further investigation.

Our research demonstrated that increased HP1a-mediated heterochromatin formation in the fat body enhanced host survival after *P. aeruginosa* infection (Fig. 1B–E, Additional file 1: Fig. S6A, B). To exclude effects of varying genetic background, HP1a was overexpressed through an RU486-inducible system in adult flies for 4 days upon eclosion. The 4-day-old adult flies were subjected to systemic *P. aeruginosa* infection and then raised with standard food without RU486 (Fig. 1D-F). Surprisingly, increased HP1-mediated heterochromatin formation in the fat body for a limited period of time during adulthood was sufficient to promote later antimicrobial responses to *P. aeruginosa* infection. These results suggest a potential prophylactic strategy using HP1a-mediated heterochromatin formation to combat *P. aeruginosa* infection.

*P. aeruginosa* is a major cause of nosocomial infections, especially in immunocompromised patients. In this study, flies immunocompromised via fat body-specific *imd* knockdown had reduced survival rates following *P. aeruginosa* infection compared to WT controls (Fig. 3D). Surprisingly, we found that concurrently increasing fat body heterochromatin formation in flies with *imd* knockdown was sufficient to rescue host resistance to WT levels (Fig. 3D). Therefore, host survival after *P. aeruginosa* infection was increased in the immunocompromised flies with concurrent fat body HP1a overexpression, indicating that, even in the immunocompromised flies, increased heterochromatin formation in the fat body can promote antimicrobial immune responses against *P. aeruginosa* infection. Interestingly, heterochromatin-mediated upregulation of AMPs upon *P. aeruginosa* infection was dramatically reduced by concurrent knockdown of *imd* in the fat body (Fig. 4A–C, F–H, Additional file 1: Fig. S8A). Importantly, we have found that *HP1a* and *imd* mRNA levels, respectively, were overexpressed and knocked down as efficiently in the *C564>HP1a* and *C564>imd*^*RNAi*^ flies with single UAS transgenes, as in *C564>HP1a; imd*^*RNAi*^ flies with two UAS transgenes (Fig. 3B, C), suggesting that *C564*-driven expression levels of UAS transgenes are not affected by the copy number of UAS transgenes in endomitotic fat body cells [54, 55]. Overall, these results suggest that heterochromatin-mediated antimicrobial responses depend partly on induction of imd-related AMPs, and also that there are likely other important immune pathway components yet to be identified. Together, those factors may be protective, even in an immunocompromised host, against *P. aeruginosa* infection. Thus, our study highlights a promising new direction for antimicrobial research in which modulation of heterochromatin formation could be used as a strategy for development of new, more effective treatments to prevent or fight *P. aeruginosa* infection.

We found that increased heterochromatin formation through *HP1a* overexpression upregulates several imd-mediated AMPs before infection (Additional file 1: Fig. S8A). To clarify the nature of the HP1a-mediated heterochromatin effect on AMPs, we investigated the expression of AMPs in the fat body with *HP1a* knockdown*.* Surprisingly, knockdown of *HP1a* by RNAi in the fat body upregulated *imd*-mediated AMPs, including *AttA*, *DptA*, and *Dro* (Additional file 1: Fig. S4C-E). Based on our results, heterochromatin levels, indicated via H3K9me2 and HP1a staining, were decreased in the early stage of *P. aeruginosa* infection (Fig. 6B–D). These results imply that decreased HP1a is required for immune responsive cells of the fat body in the early stage of *P. aeruginosa* infection to promote upregulation of AMPs genes. However, fat body-driven *HP1a* knockdown did not significantly affect host resistance against PA14 infection (Additional file 1: Fig. S4A). In contrast, increased HP1a-mediated heterochromatin formation promotes resistance to *P. aeruginosa* infection with the most notable induction of *DptA*. We analyzed HP1a binding at the *DptA* locus using chromatin immunoprecipitation (ChIP)-qPCR and found that ubiquitous HP1a overexpression increased HP1a binding at the *DptA* locus (Additional file 1: Fig. S12) [56]. Interestingly, several studies have showed that increased HP1a is associated with upregulation of euchromatic genes [57–59] and with increases of RNA polymerase pausing [60]. Together, these findings suggest that increased HP1a-mediated heterochromatin formation participates in imd-mediated *DptA* induction. Our study provides multifaceted epigenetic insights regarding HP1a-mediated antimicrobial responses during the different stage of infection.

## Conclusions

*Pseudomonas aeruginosa* is a common opportunistic pathogen associated with severe hospital-acquired infection and mortality. Effective therapeutic options for *P. aeruginosa* infection are limited due to increasing multidrug resistance. We found that increased formation of HP1a-mediated heterochromatin, an essential but poorly understood part of epigenome, promotes host survival and resistance to *P. aeruginosa* infection in *Drosophila*. Timely upregulation of heterochromatin formation in the fat body, a *Drosophila* immune organ, further increased expression of imd pathway-mediated antimicrobial peptides, with the most notable induction of Diptericin A, which is critical for HP1a-mediated antimicrobial response. Our study has identified a novel epigenetic mechanism of host immune response via heterochromatin formation, and our report highlights new therapeutic strategies to fight *P. aeruginosa* infection through heterochromatin targeting.

## Methods

### Fly strains

All fly strains were reared at 25 °C on standard yeast-based fly food, at 65% humidity, and on a 12-hour light/dark cycle. The *Oregon-R*, *w*^*1118*^, *HP1a*^*04*^, *C564-Gal4*, and *S106-gene switch-Gal4* lines were provided by the Bloomington Drosophila stock center (Bloomington, IN, USA). The *UAS-HP1a*^*RNAi*^ (31995), *UAS-HP1a*^*RNAi*^ (31994), *UAS-imd*^*RNAi*^ (9253), and *UAS-DptA*^*RNAi*^ (41284) lines were provided by the Vienna Drosophila RNAi Center (Vienna, Austria). The *UAS-DptA* (109923) line was provided by the Drosophila Genomics Resource Center (Bloomington, IN, USA). *hs-HP1a* flies were generous gifts from Dr. Lori Wallrath (University of Iowa, IA, USA) and Dr. Gunter Reuter (Martin Luther University Halle, Halle, Germany). *UAS-HP1a* flies were kind gifts from Dr. Willis Li (University of California San Diego, CA, USA). *Lsp2-Gal4* flies were a kind gift from Dr. Guang-Chao Chen (Academia Sinica, Taiwan). *UAS-Dro*, *UAS-AttA*^*RNAi*^, *iso w*^*1118*^, *Dpt*^*SK1*^, *∆AMP*^*+Dpt*^, and *∆AMP* flies were kind gifts from Dr. Mark Hanson and Dr. Bruno Lemaitre (École Polytechnique Fédérale de Lausanne, Switzerland). The *UAS-HP1a; UAS-imd*^*RNAi*^, *UAS-HP1a; UAS-DptA*^*RNAi*^, and *UAS-HP1a; UAS-AttA*^*RNAi*^ lines were constructed by chromosome recombination.

For bacterial infection experiments, unless otherwise specified, progeny from male *w*^*1118*^ flies crossed with females with the transgenic fat body-specific Gal4 driver were used as wild-type control. Progeny from UAS transgene male flies, as listed above, crossed with female flies with the transgenic fat body-specific Gal4 driver were used as the experimental group.

### Bacterial infection

*Pseudomonas aeruginosa* PA14 was stored as frozen stocks (in 50% glycerol) at – 80 °C. The bacterium was kindly gifted from Dr. Chang-Shi Chen (National Cheng Kung University, Taiwan). Before inoculation, the bacterial glycerol stock was streaked onto the LB agar plates and incubated overnight (~15 h.) at 37°C. A single bacterial colony was selected from the cultured plates and then used to inoculate 5 mL LB broth in a sterile tube. After incubation at 37°C, with shaking (250 rpm), overnight, the culture was diluted in 5 mL fresh LB media to reach OD_600_ = 0.03 and then subjected to further incubation at 37°C, with shaking at 250 rpm, until its optical density at 600 nm reached 1.6. Finally, the bacterial density was adjusted to a suitable concentration (OD_600_ = 0.1, about 108 cells/ml, OD_600_ = 0.01, or OD_600_ = 0.001) for use in inoculation throughout the study, unless otherwise specified. Inoculation was achieved by pricking the thorax of a 3–5 day old male fly with a tungsten needle (25 gauge) that had been dipped into an inoculum [61]. Infected flies were incubated in vials (10 flies/vial) at 22 °C, at 60% humidity, on a 12-h light/dark cycle. The LINKO LKU-6000 spectrophotometer was used to quantify bacterial concentration at 600 nm. Survival rate after bacterial infection was determined by counting live flies every 3 h.

### Bacterial quantification

Flies were homogenized in 200 μl phosphate-buffered saline (PBS) and homogenates were diluted (× 10) appropriately and then plated on LB agar plates. Colony-forming units (CFU) were counted as a measure of bacterial load in the flies.

### Gene switch inducible system

To induce the expression of specific genes, adult flies were provided food containing 600 μM RU486 and EtOH (control) at 0 day old. After 4 days, flies were inoculated with bacteria.

### qRT-PCR

Fat body tissues from four flies were collected at DNA/RNA Shield™ (ZYMO RESEARCH, USA) and mRNA was extracted using *Quick*-RNA™ Microprep Kit (ZYMO RESEARCH, USA). cDNA synthesis was performed using the PrimeScript RT reagent Kit (TaKaRa, Japan). RT-PCR was performed in triplicate for each sample using SYBR Green PCR Master Mix (Applied Biosystems, USA) on the Thermo Fisher Scientific StepOne system. Relative levels of target gene mRNA were normalized to the reference gene *rpl32*. The following primers were used:

**Table:** 

Gene	Forward (5′->3′)	Reverse (5′->3′)
*rpl32*	GCTAAGCTGTCGCACAAATG	GTTCGATCCGTAACCGATGT
*HP1a*	CGCAAGGATGAGGAGAAGTCA	TCCTGAAACGGGAATGGTGTC
*imd*	GGCATGTCGGAAGGACAGAT	GCTGTTTGTCTTGCGCTTCT
*DptA*	GCTGCGCAATCGCTTCTACT	TGGTGGAGTGGGCTTCATG
*Dro*	CCACCACTCCAAGCACAATGAA	CATCTTTAGGCGGGCAGAATGG
*AttA*	CTCCTGCTGGAAAACATC	GCTCGTTTGGATCTGACC
*AttB*	GGGTAATATTTAACCGAAGT	GTGCTAATCTCTGGTCATC
*AttC*	CTGCACTGGACTACTCCCACATCA	CGATCCTGCGACTGCCAAAGATTG
*Bara*	GGTGAGCATGTGTACACCGA	GGCGGAAAAATTGGGACCAC
*BomS1*	GCCAATGCTGTTCCACTGTC	GGGTTGAAACTTCCTACTTGCC
*CecA1*	ATCAGTCGCTCAGACCTCAC	GATTGTGGCATCCCGAGTGT
*CecC*	CAATCGGAAGCCGGTTGGCTG	GCGCAATTCCCAGTCCTTGAATGG
*Def*	GTTCTTCGTTCTCGTGG	CTTTGAACCCCTTGGC
*Drs*	CGTGAGAACCTTTTCCAATATGATG	TCCCAGGACCACCAGCAT
*Dso1*	AACCTGGAACCGTGCTCATC	GGCAAATGTCAAATGTTGGGTC
*Dso2*	CTTCGCTCTGATTGCGGCTT	AATCAACGTGTGTCCGCCA
*Mtk*	AACTTAATCTTGGAGCGA	CGGTCTTGGTTGGTTAG
*TotA*	CCCTGAGGAACGGGAGAGTA	CTTTCCAACGATCCTCGCCT
*STAT92E*	CTGGGCATTCACAACAATCCAC	GTATTGCGCGTAACGAACCG

### Immunostaining

Adult fat bodies with attached dorsal cuticles were dissected from the fly abdomen in PBS. The fat body tissues were fixed with 4% paraformaldehyde in PBS for 15 min at RT followed by washing three times for 10 min with PBST (PBS containing 0.1% Triton X-100). The fat body from each fly was incubated with primary antibodies in PBST with normal goat serum (NGS) at 4 °C for 48 h. The tissues were incubated with primary antibodies, including mouse monoclonal anti-HP1a (C1A9, 1:300; Developmental Studies Hybridoma Bank) and/or rabbit anti-H3K9me2 (07-212, 1:500; Upstate Biotechnology) in PBST with NGS. After washing three times in PBST for 10 min, each tissue was incubated with secondary antibodies and DNA staining dye (Hoechst, 1:1000; Invitrogen) at 4 °C for 24 h. The tissues were incubated with secondary antibodies, including goat anti-mouse conjugated to Alexa Fluor-594 (Thermo Fisher Scientific, USA) and/or goat anti-rabbit conjugated to Alexa Fluor-647 (Thermo Fisher Scientific) in PBST with NGS. Tissues were then washed 3 times in the PBST for 10 min, and the fat body tissues were then dissected in PBST and mounted on slides with VECTASHIELD® antifade mounting medium (Vector Laboratories, USA). Images were captured using a confocal microscope (Carl Zeiss LSM780, Core Facility Center, National Cheng Kung University, Tainan, Taiwan).

### Chromatin immunoprecipitation

For ChIP experiments, 120 male flies were collected to enable ChIP using Magna ChIP™ HiSens Kit (Merck Millipore, USA). The flies were fixed with 0.5% formaldehyde for 10 min at RT. The crosslinked chromatin was stopped by glycine and washing with ice cold PBS buffer. The tissue lysate was then sonicated to shear DNA into ~200-1000 bp using sonicator (LINKO Ultrasonic processor UP-300). Sheared crosslinked chromatin was incubated with 2.5 μl HP1a antibody (C1A9; Developmental Studies Hybridoma Bank) or 2.5 μg control IgG (#5415; Cell Signaling) at 4 °C overnight. RT-PCR was performed in triplicate for each sample using SYBR Green PCR Master Mix (Applied Biosystems, USA) on the Thermo Fisher Scientific StepOne system. The following ChIP primers for qPCR were used:

**Table:** 

Gene	Forward (5′->3′)	Reverse (5′->3′)
*cdc2*	CCATATGTGCCCTCGCCAAT	GTAGCTAGCTTAGCATCGTT
*rp49*	GTAAACGCGGTTCTGCATGAGC	ATCGGTTACGGATCGAACAAGC
*DptA-1*	ATGAGACAATAACCGCCGTAGG	CAAAGTAAGGCGACGGCAAT
*DptA-2*	CATTGCCGTCGCCTTACTTT	TTCAGTCCAATCTCGTGGCG
*DptA-3*	ACAATGGACGCCACGAGATT	AGCTAGACTCGGATACCAATCG
*DptA-4*	AAGTGGGAAGCACCTACACCT	GTTCCGGGTTAAACAAACAACGC

### Statistical analysis

All data were analyzed and presented using PRISM 6 (GraphPad, San Diego, CA). Statistical analyses were performed using Student's t-test and reported as mean ± SD, as specified in the respective figure legends. Significance levels are indicated as *p*-values below 0.05. All survival data were analyzed using the Log-rank (Mantel-Cox) test.

## Supplementary Information

**Additional file 1: Figure S1.** HP1a-mediated heterochromatin formation is imperative to regulate resistance to systemic *P. aeruginosa* infection in *Drosophila*. **Figure S2.** Other independent experiments also show that increased HP1a-mediated heterochromatin formation in the fat body promotes resistance against systemic *P. aeruginosa* PA14 infection, related to Fig. 1. **Figure S3.** Increased fat body HP1a-mediated heterochromatin promotes host resistance to PA14 infection with a lower bacterial dose. **Figure S4.** HP1a knockdown in the fat body does not affect survival after systemic *P. aeruginosa* infection. **Figure S5.** RU486 induces fat body-specific *S106-Gene Switch*-driven gene expression, as demonstrated by GFP expression. **Figure S6.** Increased heterochromatin formation in the fat body promotes survival and host resistance to *P. aeruginosa* PA14 infection. **Figure S7.** CFU measurements from fat body-driven HP1a and control flies after *P. aeruginosa* PA14 infection. **Figure S8.** Increased heterochromatin formation upregulates a broad spectrum of AMPs, and upregulation of *AttA*, *DptA*, and *Dro* is heavily dependent on the imd pathway. **Figure S9.** Increased heterochromatin formation in the fat body promotes upregulation of imd-mediated AMPs in the middle stage of *P. aeruginosa* infection. **Figure S10.** Validation and effects of AMP gene expression in the fat body. **Figure S11.***DptA* and other AMPs, in combination, are required for host resistance to PA14 infection. **Figure S12.** Increased HP1a-mediated heterochromatin formation leads to more HP1a binding at the *DptA* locus.

## Data Availability

All data generated or analyzed during this study are included in this published article and its supplementary information files.
