# Supplementary material for: HP1a-mediated heterochromatin formation promotes antimicrobial responses against Pseudomonas aeruginosa infection
Source: BMC Biol. 2022 Oct 20;20:234. doi: 10.1186/s12915-022-01435-8 (PMC9583553; doi:10.1186/s12915-022-01435-8)
Supplement: Supplementary file 1 — Additional file 1: Figure S1. HP1a-mediated heterochromatin formation is imperative to regulate resistance to systemic P. aeruginosa infection in Drosophila. Figure S2. Other independent experiments also show that increased HP1a-mediated heterochromatin formation in the fat body promotes resistance against systemic P. aeruginosa PA14 infection, related to Fig. 1. Figure S3. Increased fat body HP1a-mediated heterochromatin promotes host resistance to PA14 infection with a lower bacterial dose. Figure S4. HP1a knockdown in the fat body does not affect survival after systemic P. aeruginosa infection. Figure S5. RU486 induces fat body-specific S106-Gene Switch-driven gene expression, as demonstrated by GFP expression. Figure S6. Increased heterochromatin formation in the fat body promotes survival and host resistance to P. aeruginosa PA14 infection. Figure S7. CFU measurements from fat body-driven HP1a and control flies after P. aeruginosa PA14 infection. Figure S8. Increased heterochromatin formation upregulates a broad spectrum of AMPs, and upregulation of AttA, DptA, and Dro is heavily dependent on the imd pathway. Figure S9. Increased heterochromatin formation in the fat body promotes upregulation of imd-mediated AMPs in the middle stage of P. aeruginosa infection. Figure S10. Validation and effects of AMP gene expression in the fat body. Figure S11.DptA and other AMPs, in combination, are required for host resistance to PA14 infection. Figure S12. Increased HP1a-mediated heterochromatin formation leads to more HP1a binding at the DptA locus. [file 12915_2022_1435_MOESM1_ESM.docx]

**Supplementary Information for**

**Research article**

**HP1a-mediated heterochromatin formation promotes antimicrobial responses against *Pseudomonas aeruginosa* infection**

**Po-Jen Wu^1,2^,** **Shian-Jang Yan^*,1,2^**

***** Correspondence: johnyan@gs.ncku.edu.tw

1. Institute of Basic Medical Sciences, College of Medicine, National Cheng-Kung University, No. 1, University Road, Tainan City, Taiwan
2. Department of Physiology, College of Medicine, National Cheng-Kung University, No. 1, University Road, Tainan City, Taiwan

**This file includes:**

Figures S1 to S12

**
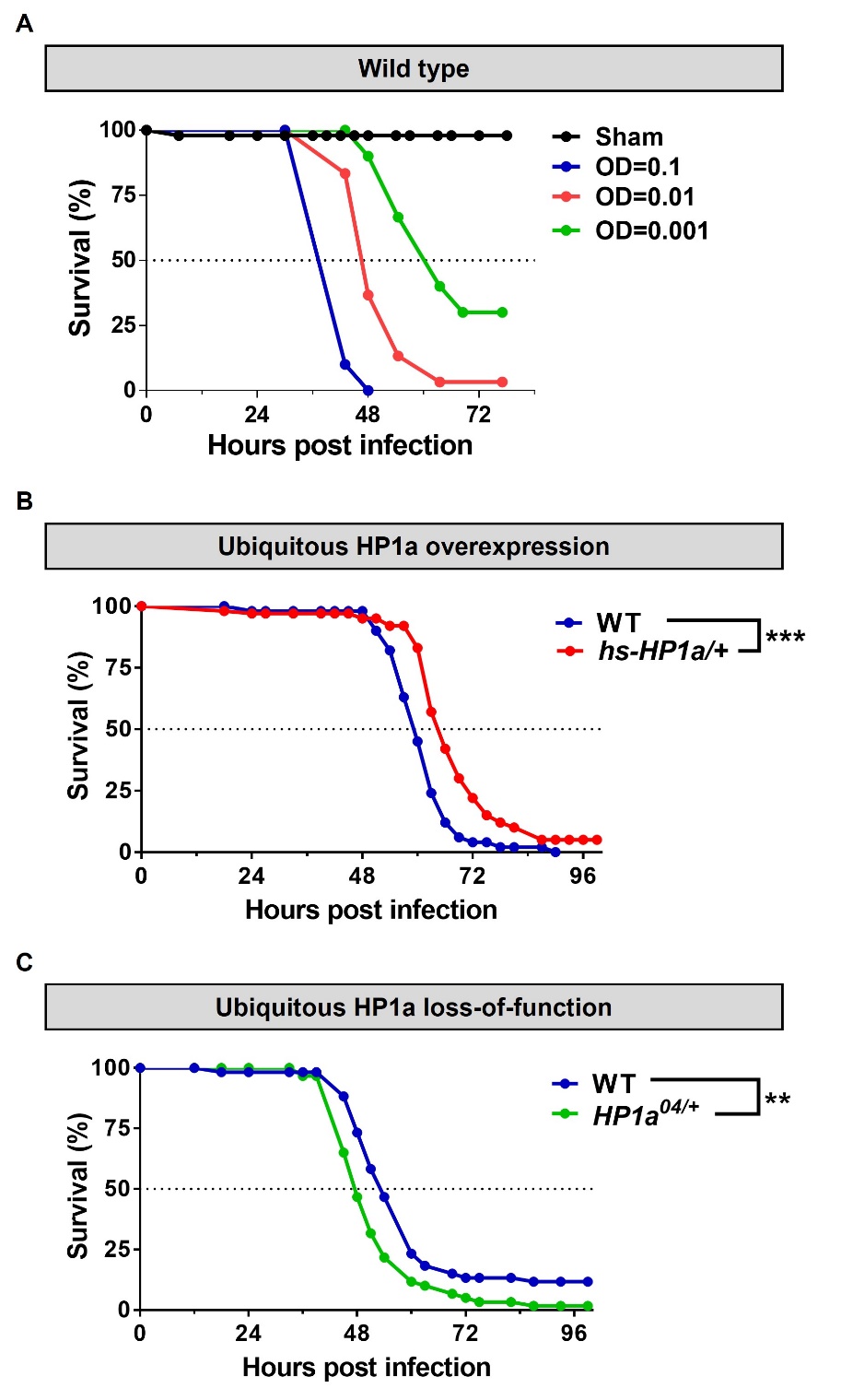
**

**Fig. S1. HP1a-mediated heterochromatin formation is imperative to regulate resistance to** **systemic *P. aeruginosa* infection in *Drosophila*.**

**A** Inoculation with *P. aeruginosa* PA14 dose-dependently increased the mortality of flies. The sham group was pricked with phosphate-buffered saline (PBS). Sham, n=99; OD=0.1, n=20; OD=0.01, n=30; OD=0.001, n=30. **B** *hs-HP1a/+* (progeny from male *hs-HP1a* flies crossed with *w^1118^* females) flies with ubiquitous expression of a 20% higher level of HP1a compared to wild type (WT, progeny from male *Oregon-R* flies crossed with *w^1118^* females) flies showed significantly higher rates of survival than WT upon PA14 infection. WT, n=49; *hs-HP1a*/+, n=60. **C** *HP1a^04/+^* (progeny from male *HP1a^04^* flies crossed with *w^1118^* females) flies with ubiquitous expression of 50% less HP1a showed significantly lower rates of survival than WT upon PA14 infection. WT, n=60; *HP1a^04/+^*, n=60. **p < 0.01, ***p < 0.001 by log-rank test. Inoculum of OD_600_=0.1.

**
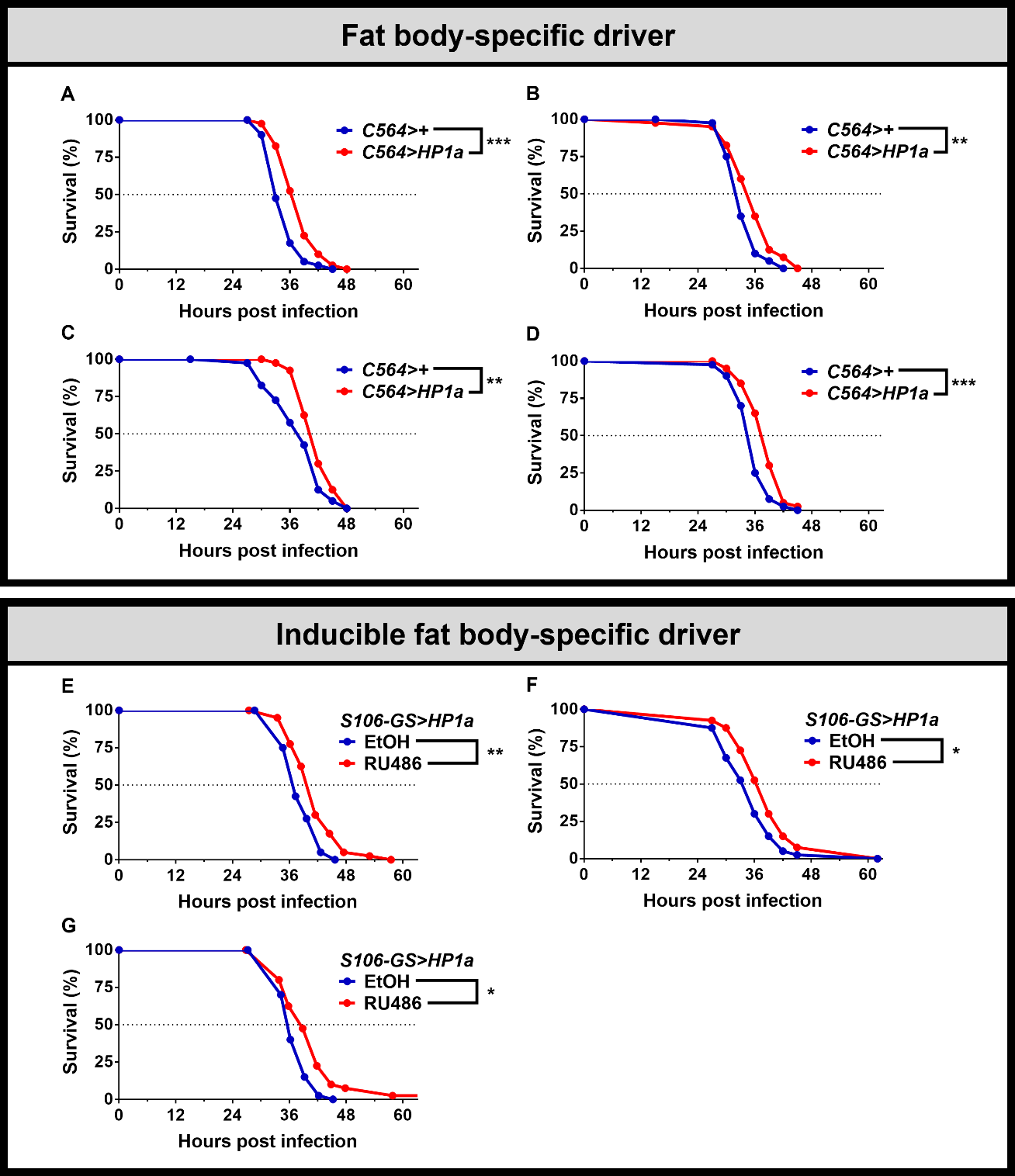
**

**Fig. S2. Other independent experiments** **also show that increased HP1a-mediated heterochromatin formation in the fat body promotes resistance against systemic *P. aeruginosa* PA14 infection, related to Figure 1.**

**A-D** The survival curve of flies with fat body-specific *C564*-driven *HP1a* overexpression, compared to control, after PA14 infection. **E-G** The survival curve of flies with RU486-inducible fat body specific *HP1a* overexpression after PA14 infection. Control and treated flies received food with either EtOH (vehicle) or RU486, respectively, for 4 days upon eclosion. n=40 flies/group. *p < 0.05, **p < 0.01, ***p < 0.001 by log-rank test. Inoculum of OD_600_=0.1.


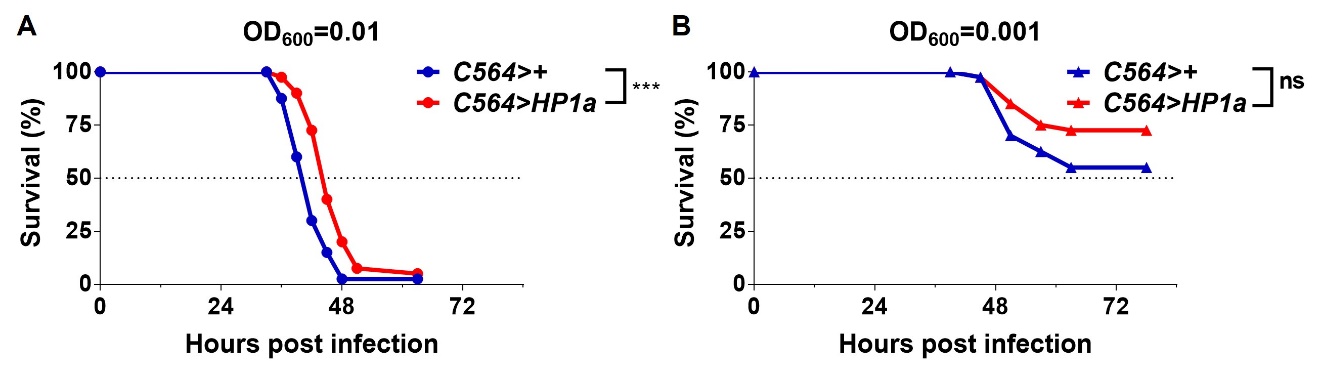


**Fig. S3. Increased fat body HP1a-mediated heterochromatin promotes host resistance to PA14 infection with a lower bacterial dose.**

Inoculation of *P. aeruginosa* PA14 OD_600_=0.01 (**A**) and 0.001 (**B**) in flies with fat body-specific *C564*-driven HP1a overexpression compared to control. n=40 flies/group. ***p < 0.001 by log-rank test. ns, not significant.

**
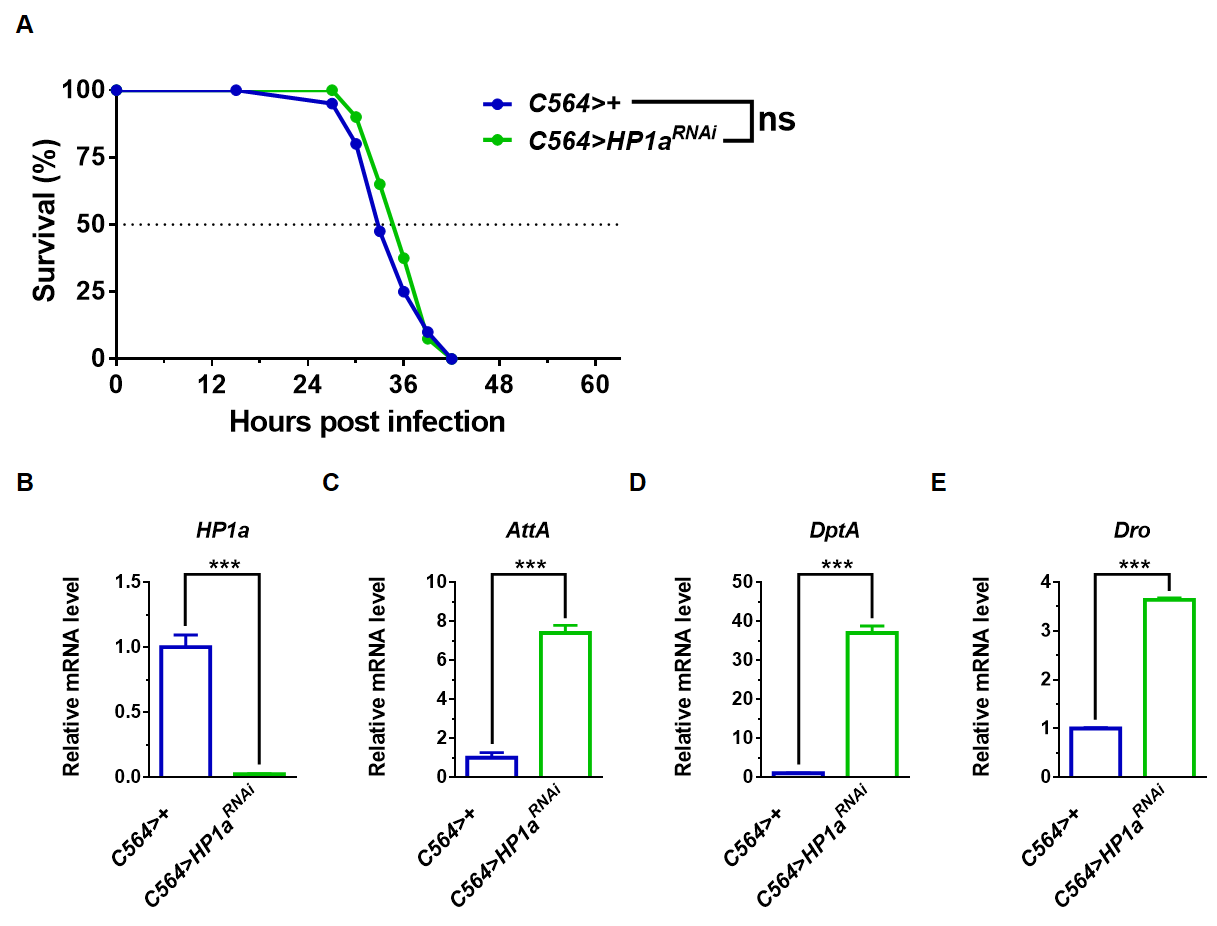
**

**Fig. S4. HP1a knockdown in the fat body does not affect survival after systemic *P. aeruginosa* infection.**

**A** There was no significant difference in survival of flies with fat body-specific *C564*-driven HP1a knockdown (BDSC 31994) compared to control after PA14 infection. n=40/group. Inoculum of OD_600_=0.1. ns, not significant by log-rank test. **B-E** Relative expression of *HP1a* (**B**) and AMP (**C-E**) mRNA in the fat body was measured before PA14 infection. n=6 flies/group. Data are shown as mean ± SD and ***p < 0.001 by Student’s t-test. ns, not significant.


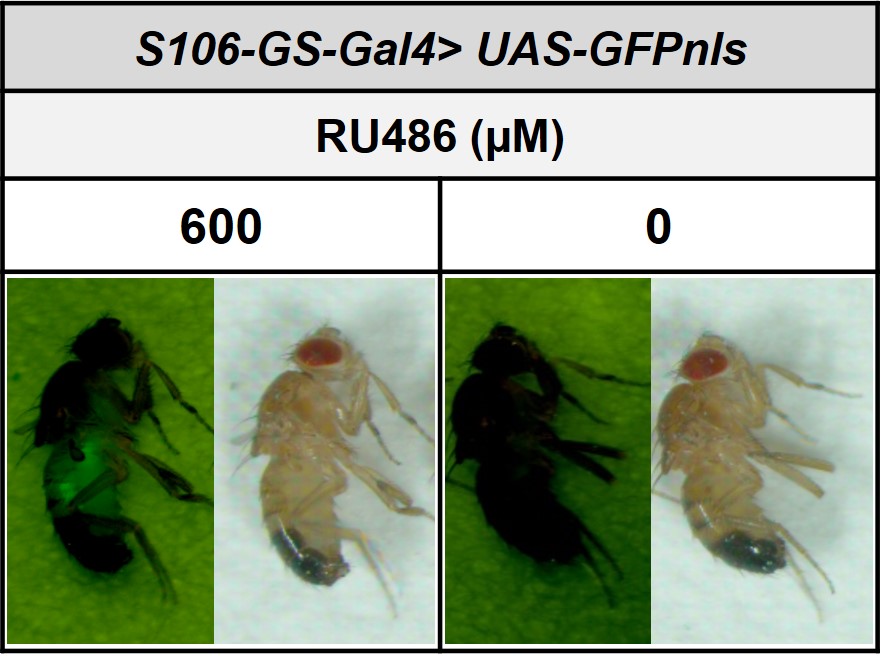


**Fig. S5. RU486 induces fat body-specific *S106-Gene Switch*-driven gene expression, as demonstrated by GFP expression.**


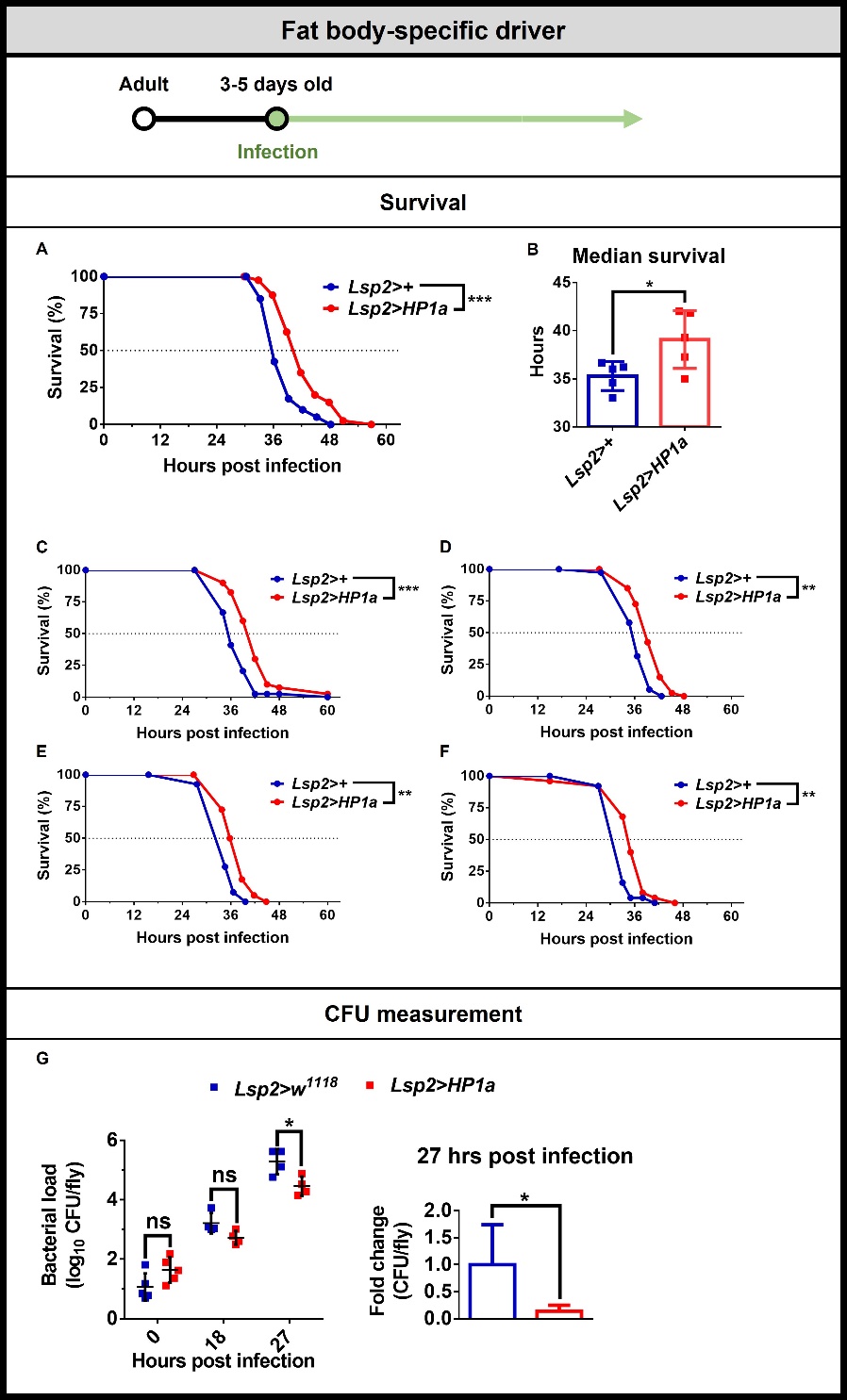


**Fig. S6. Increased heterochromatin formation in the fat body promotes survival and host resistance to *P. aeruginosa* PA14 infection.**

**A, B** The survival curve and median survival in flies with fat body-specific *Lsp2*-driven *HP1a* overexpression compared to control after PA14 infection. n=40 flies/group. ***p < 0.001 by log-rank test. For median survival, data are shown as mean ± SD from 5 independent experiments and *p < 0.05 by Student’s t-test. **C-F** The other 4 independent experiments of flies with fat body-specific *Lsp2*-driven *HP1a* overexpression, compared to control, after PA14 infection. n=38-40 flies/group (**C-E**). n=25 flies/group (**F**). For the survival curve, **p < 0.01 and ***p < 0.001 by log-rank test. **G** Bacterial loads of *Lsp2*-driven HP1a and control flies were counted at 0, 18, and 27 hpi. The CFU experiments at 18 and 27 hpi are representative of 4 independent experiments, and each experiment included 5 flies for CFU measurement. CFU experiments at 0 hpi are representative of 5 independent experiments, and each experiment included 1 fly for the CFU measurement. *p < 0.05 by Student’s t-test. CFU, colony forming unit; hpi, hrs post infection; ns, not significant.

**
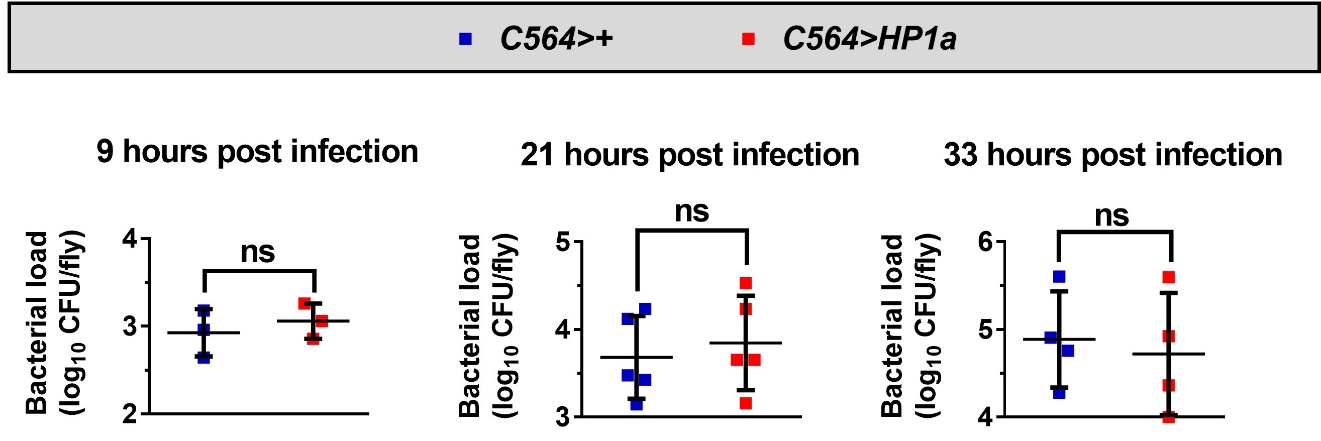
**

**Fig. S7. CFU measurements from fat body-driven HP1a and control flies after *P. aeruginosa* PA14 infection.**

Bacterial loads of *C564*-driven HP1a and control flies were counted at 9, 21, and 33 hpi. The CFU measurements after infection are representative of 3-5 independent experiments, and each experiment included measurements from 5 flies. ns, not significant by Student’s t-test.


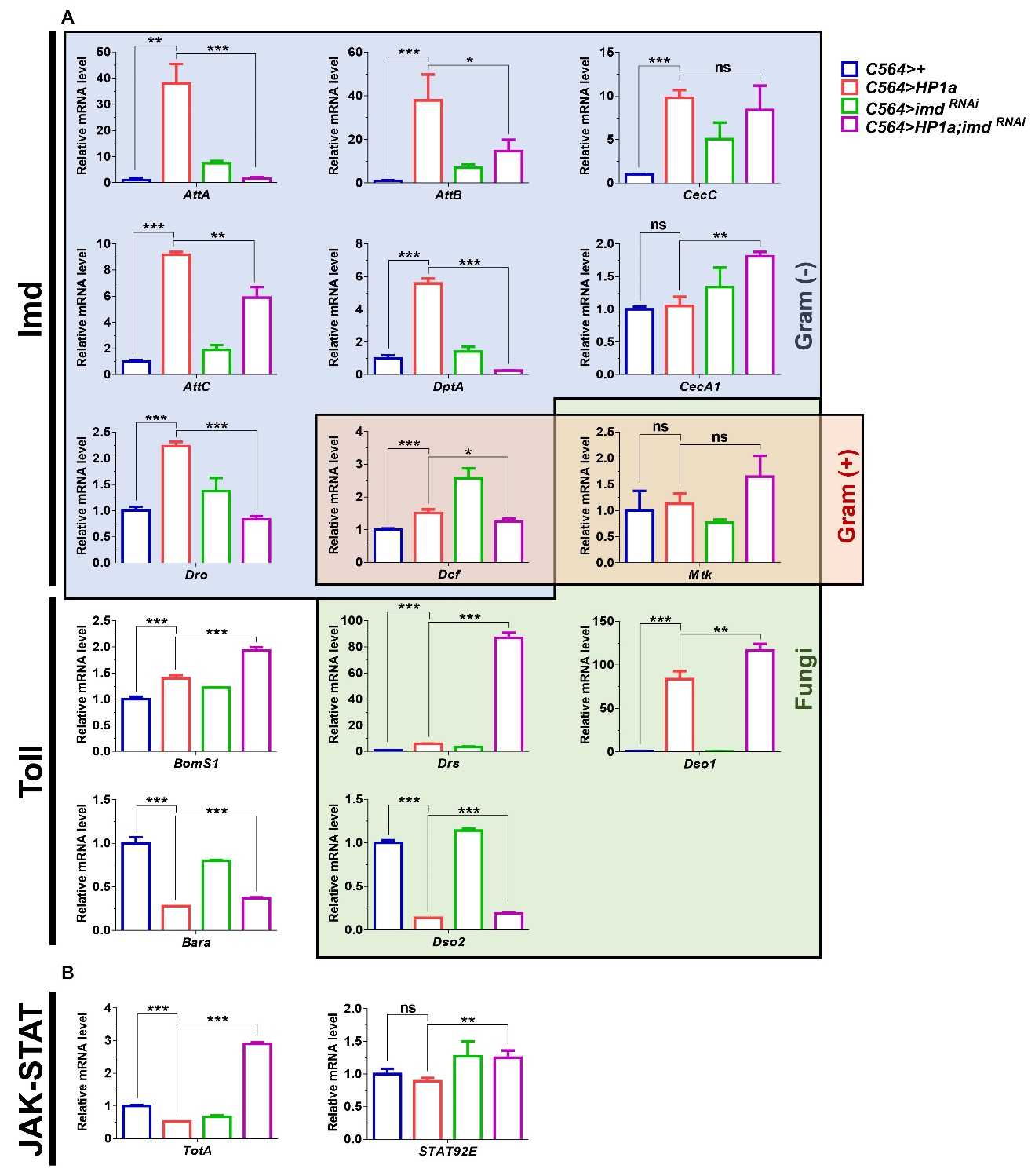


**Fig. S8. Increased heterochromatin formation upregulates a broad spectrum of AMPs, and upregulation of *AttA*, *DptA*, and *Dro* is heavily dependent on the imd pathway.**

Assessment of relative mRNA expression of AMPs (**A**) and JAK-STAT response genes (**B**) in the fat body were performed before PA14 infection. n=8 flies/group. Data are shown as mean ± SD and *p < 0.05, **p < 0.01, ***p < 0.001 by Student’s t-test. AttA, Attacin-A; AttB, Attacin-B; AttC, Attacin-C; Bara, Baramicin; BomS1, Bomanin Short 1; CecA1, Cecropin A1; CecC, Cecropin C; Def, Defensin; DptA, Diptericin A; Dro, Drosocin; Drs, Drosomycin; Dso1, Daisho1 ; Dso2, Daisho2; Mtk, Metchnikowin; STAT92E, Signal-transducer and activator of transcription protein at 92E; TotA, Turandot A; ns, not significant.


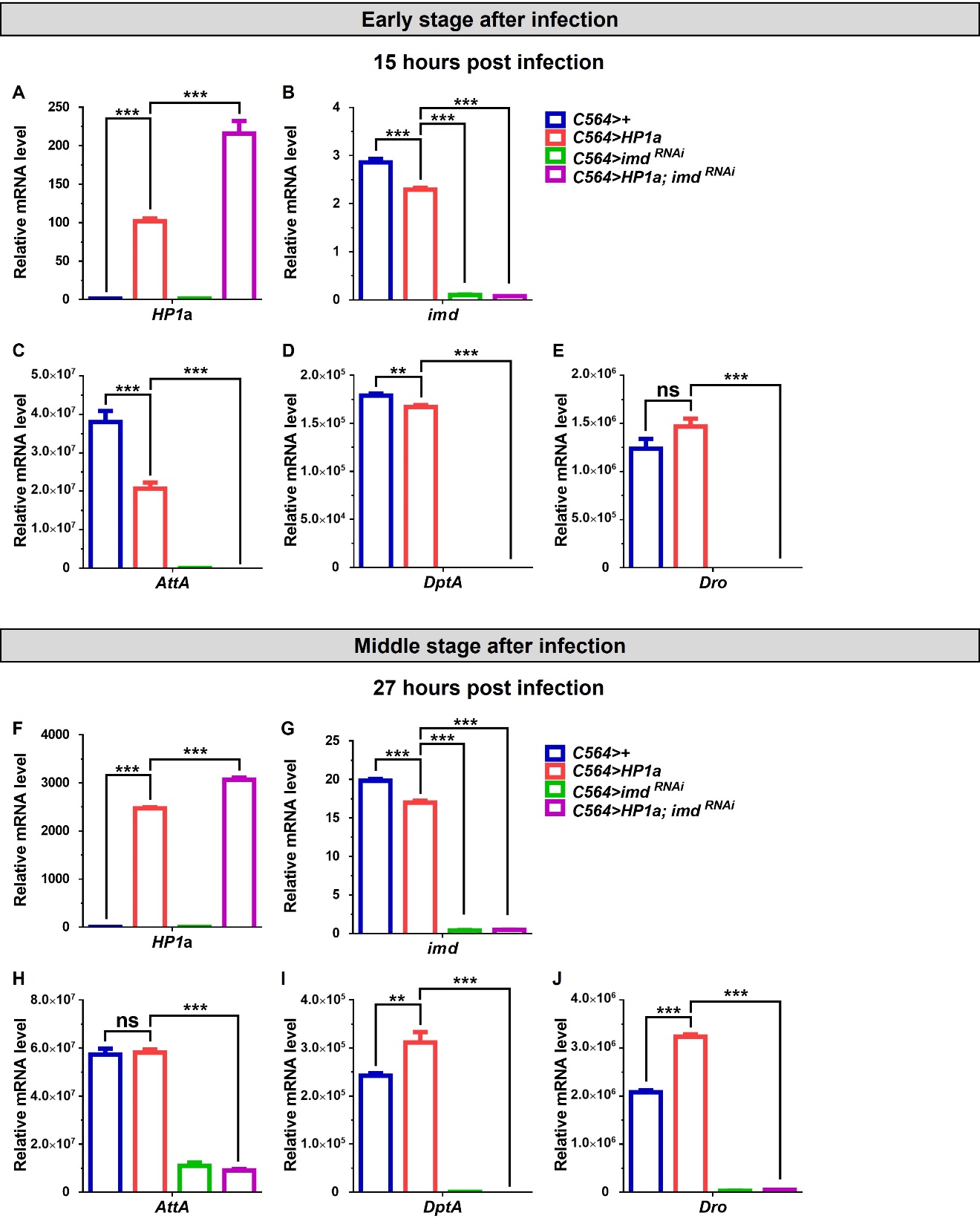


**Fig. S9. Increased heterochromatin formation in the fat body promotes upregulation of imd-mediated AMPs in the middle stage of *P. aeruginosa* infection.**

Relative expression of *HP1a*, *imd*, *DptA*, *Dro*, and *AttA* mRNA in the fat body was measured in the early (**A-E**) and middle (**F-J**) stages of PA14 infection. n=4 flies/group at each time point. Data are shown as mean ± SD and **p < 0.01, ***p < 0.001 by Student’s t-test. ns, not significant.

**
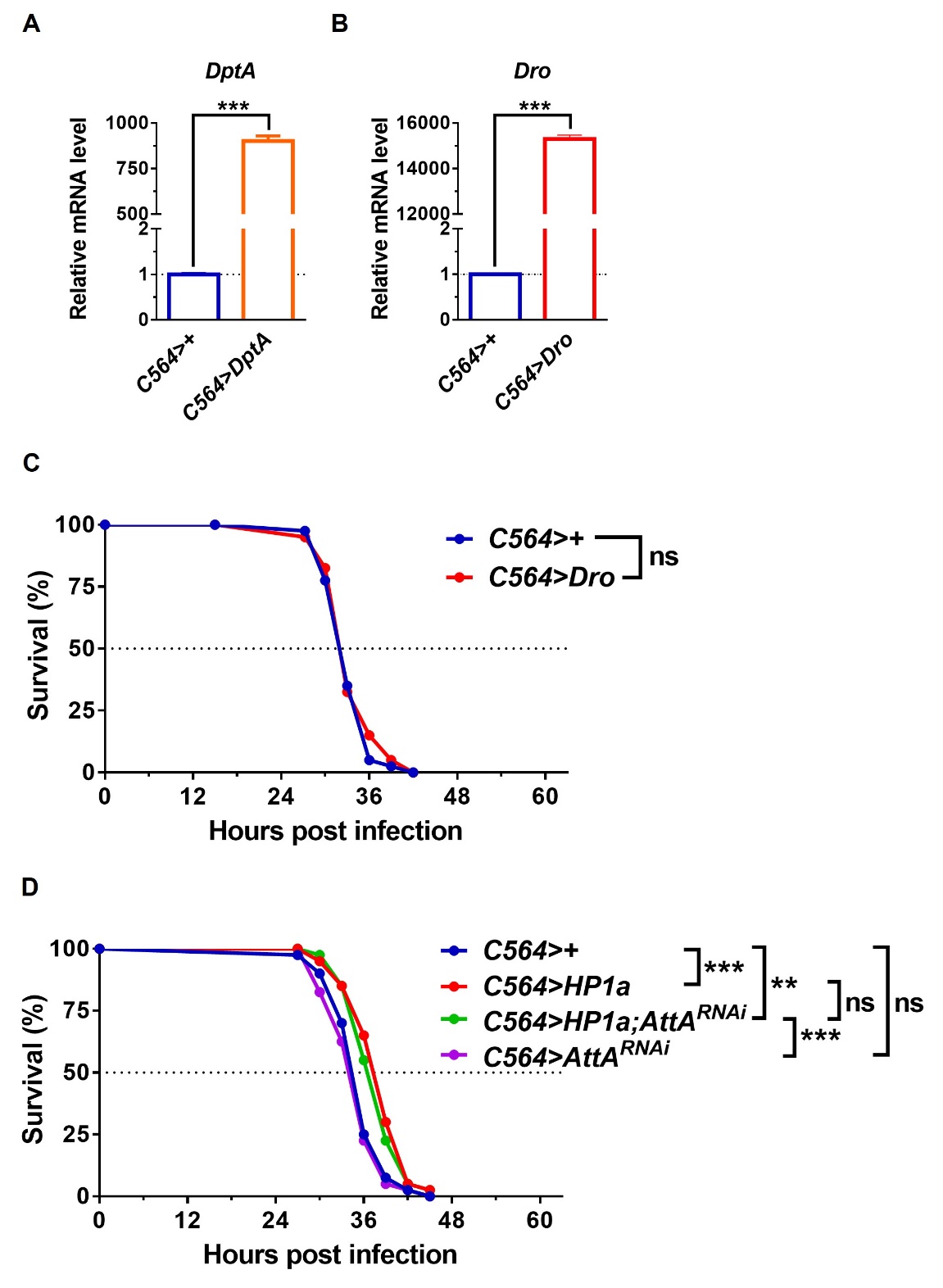
**

**Fig. S10. Validation and effects of AMP gene expression in the fat body.**

**A, B** Relative expression of *DptA* (**A**) and *Dro* (**B**) mRNA in the fat body was measured before PA14 infection. n=8 flies/group. Data are shown as mean ± SD and ***p < 0.001 based on Student’s t-test. **C** *Dro* overexpression is not sufficient to promote host resistance to PA14 infection. n=40/group. **D** *AttA* is not necessary to promote host resistance to PA14 infection. n=40/group. Inoculum of OD_600_=0.1. For bacterial infection experiments (**C, D**), **p < 0.01 and ***p < 0.001 by log-rank test. ns, not significant.

**
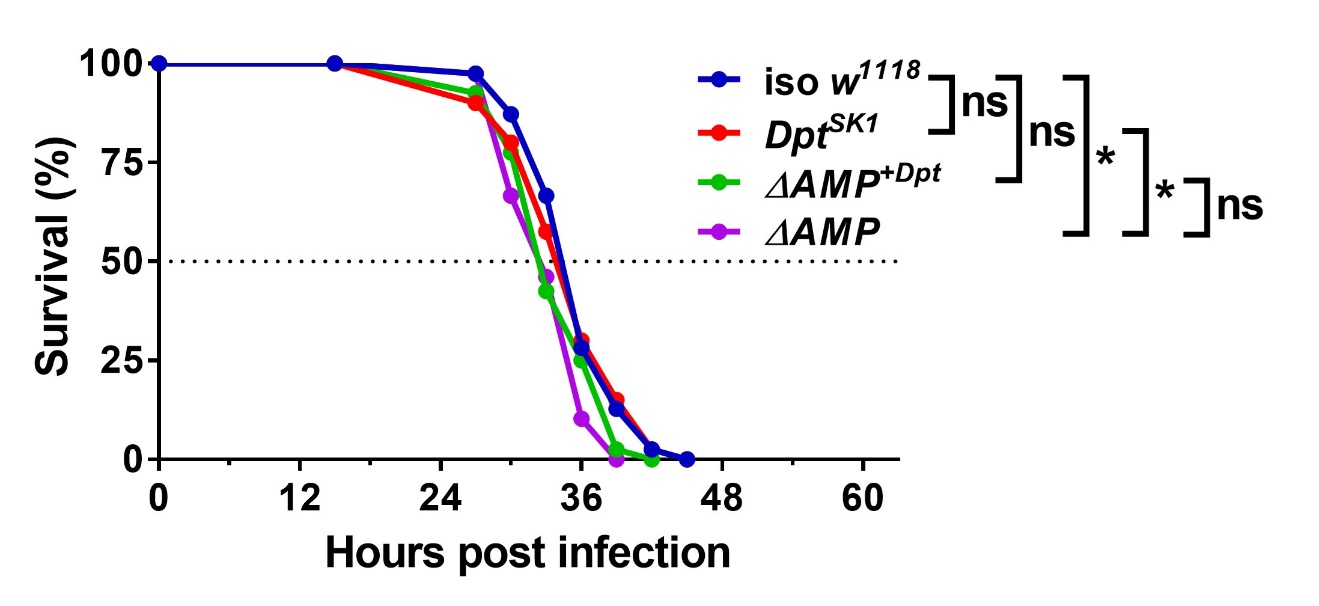
**

**Fig. S11. *DptA* and other AMPs, in combination, are required for host resistance to PA14 infection.**

The survival curve in flies with combined AMP mutations. Flies lacking 10 AMPs (*AttA-D, Def, DptA-B, Dro, Drs*, and *Mtk* mutations), *∆AMP* (n=40), were more vulnerable to PA14 infection. The *DptA-B* mutants, *Dpt^SK1^* (n=40), and mutants with loss of 8 AMPs (*AttA-D, Def, Dro, Drs*, and *Mtk* mutations) except *DptA-B,* *∆AMP^+Dpt^* (n=40), did not affect host susceptibility to PA14 infection compared to control, iso *w^1118^* (n=39). *p < 0.05 by log-rank test. Inoculum of OD_600_=0.1. AttA, Attacin-A; AttB, Attacin-B; AttC, Attacin-C; AttD, Attacin-D; Def, Defensin; DptA, Diptericin A; DptB, Diptericin B; Dro, Drosocin; Drs, Drosomycin; Mtk, Metchnikowin; ns, not significant.

**
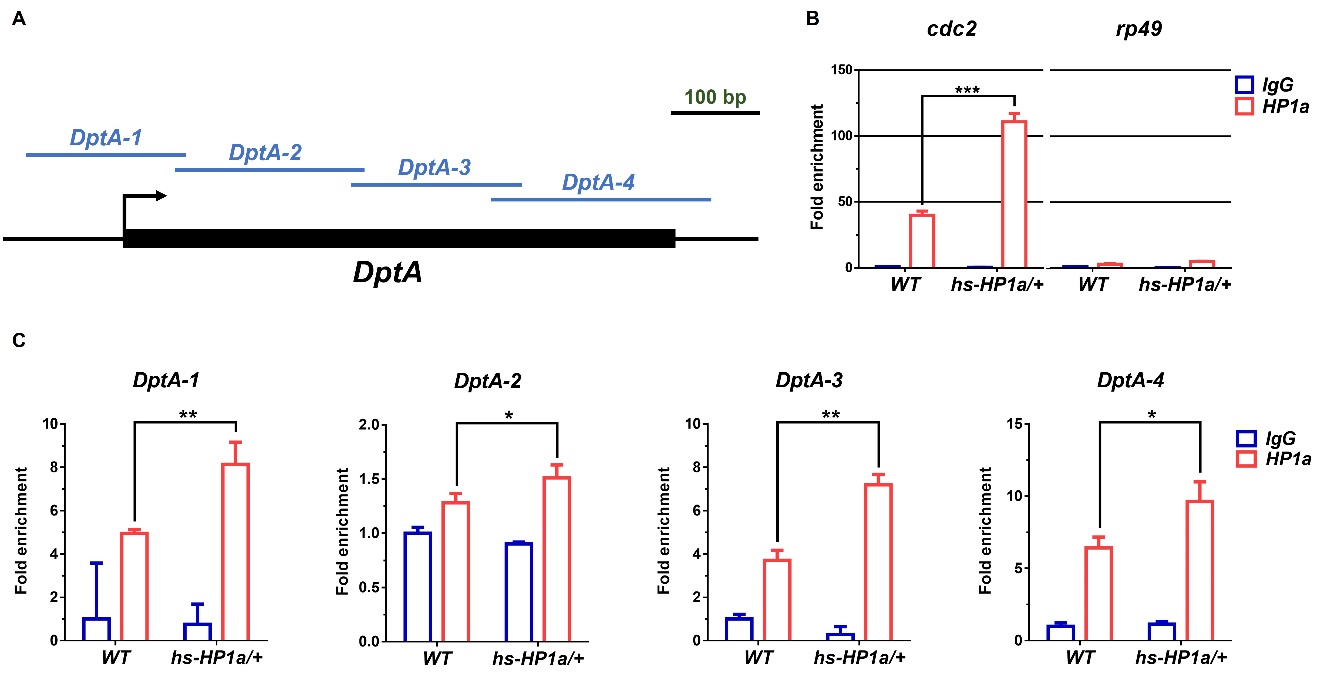
**

**Fig. S12. Increased HP1a-mediated heterochromatin formation leads to more HP1a binding at the *DptA* locus.**

**A** Four sets of primers corresponding to *DptA* in the transcription start site (arrow) and gene body (black box) were used to amplify immunoprecipitated DNA. **B, C** Chromatin immunoprecipitation (ChIP) analyses of *DptA* from male WT and *hs-HP1a/+* flies (n=120). Chromatin was immunoprecipitated with antibodies to HP1a and IgG (negative control). *rp49* primers were used as a negative control and *cdc2* primers as a positive control. The fold enrichment represents the relative amount of qPCR products. Data are shown as mean ± SD and *p < 0.05, **p < 0.01, ***p < 0.001 by Student’s t-test. WT, wild type.
